# A Multi-Omics Analysis of Recombinant Protein Production in Hek293 Cells

**DOI:** 10.1371/journal.pone.0043394

**Published:** 2012-08-24

**Authors:** Stefanie Dietmair, Mark P. Hodson, Lake-Ee Quek, Nicholas E. Timmins, Peter Gray, Lars K. Nielsen

**Affiliations:** Australian Institute for Bioengineering and Nanotechnology (AIBN), The University of Queensland, Brisbane, Queensland, Australia; Technical University of Denmark, Denmark

## Abstract

Hek293 cells are the predominant hosts for transient expression of recombinant proteins and are used for stable expression of proteins where post-translational modifications performed by CHO cells are inadequate. Nevertheless, there is little information available on the key cellular features underpinning recombinant protein production in Hek293 cells. To improve our understanding of recombinant protein production in Hek293 cells and identify targets for the engineering of an improved host cell line, we have compared a stable, recombinant protein producing Hek293 cell line and its parental cell line using a combination of transcriptomics, metabolomics and fluxomics. Producer cultures consumed less glucose than non-producer cultures while achieving the same growth rate, despite the additional burden of recombinant protein production. Surprisingly, there was no indication that producer cultures compensated for the reduction in glycolytic energy by increasing the efficiency of glucose utilization or increasing glutamine consumption. In contrast, glutamine consumption was lower and the majority of genes involved in oxidative phosphorylation were downregulated in producer cultures. We observed an overall downregulation of a large number of genes associated with broad cellular functions (e.g., cell growth and proliferation) in producer cultures, and therefore speculate that a broad adaptation of the cellular network freed up resources for recombinant protein production while maintaining the same growth rate. Increased abundance of genes associated with endoplasmic reticulum stress indicated a possible bottleneck at the point of protein folding and assembly.

## Introduction

Recombinant proteins such as hormones, growth factors, cytokines and monoclonal antibodies play an important role in modern medicine, being used to treat a variety of diseases (e.g. diabetes, anaemia, hepatitis and cancer) [1]. Many of these proteins require a range of post-translational modifications (e.g., glycosylation, phosphorylation) to ensure correct folding, activity, safety and stability, and are therefore produced in mammalian cells [2].

The most popular mammalian host cells for the production of biopharmaceuticals are CHO cells due to their extensive characterization and history of regulatory approvals. However, CHO cells cannot perform all types of human glycosylation as they lack certain sugar transferring enzymes such as α(2–6) sialyltransferase and α(1–3/4) fucosyltransferases [3]. In addition, CHO cells are known to add potentially immunogenic glycan structures, which can result in increased clearance of the drug and reduced efficacy [4].

For these reasons, it is often advantageous and sometimes essential to produce certain recombinant proteins in human cells such as human fibrosarcoma (HT-1080), human retinal (PerC.6) or human embryonic kidney 293 cells (Hek293). One such example is Xigris (activated protein C), which is produced in Hek293 cells as the post-transitional modifications performed by CHO cells were found to be inadequate [4].

In addition to being a stable host for production of several protein therapeutics, Hek293 is the predominant cell line for transient expression of recombinant proteins [5], [6]. Transient transfection allows rapid production of recombinant proteins, but product titres are generally lower than those achieved with stably transfected cell lines [5]. If transient product titres were to be increased to the same level as stable cell lines, it could be envisaged that transient transfections may be a viable alternative to the time and labour intensive generation of stable cell lines [7]. While significant effort has been placed on optimising expression vectors, transfection protocols and media composition [5], [7]–[9], less effort has been placed on understanding which cellular features are required for high productivity in Hek293 cells and subsequent engineering of an improved host cell.

Transient systems are difficult to study due to their nature, but in many cases strategies known to enhance cell specific productivities of stable cell lines (e.g., cultivation at lower temperatures, hyperosmolarity, addition of sodium butyrate, expression of cell cycle regulators) were shown to increase transient product titres [6], [10]–[13]. Thus, it appears that factors influencing productivity in stable and transient cell lines are similar.

To pave the way for engineering of Hek293 cells with improved protein production capacity in a transient and stable setting, we sought to gain a better understanding of the cellular mechanics underlying high productivity in Hek293 cells. Therefore, we have compared a stable Hek293 cell line producing a heavy chain variable region fused to the Fc region of a human IgG (dAb-Fc), and its non-producing parental cell line using a range of omics technologies. Triplicate bioreactor cultures were performed for each cell line and samples for analysis of the transcriptome, metabolome and fluxome were taken during exponential phase. This multi-omics approach allowed extensive characterization of producer and non-producer cultures and identified several potential avenues for cellular engineering.

## Materials and Methods

### Cell Culture

Hek293F cells (Invitrogen, Carlsbad, CA) were cultivated in Hek293 Freestyle Expression Medium (Invitrogen) supplemented with 0.5 mM glutamine, 3 mM Glutamax, 100 µg/mL dextran sulfate (M_w_ = 5000 Da) and 4 mL/L Pluronic-F12. Cells were cultivated in vented shake flasks (Corning, NY, USA) in a Multitron humidified shaking incubator (Infors HT, Basel, Switzerland) set to 37°C, 5% CO_2_, and 170 rpm. Using a liposome based transfection method, Hek293F cells were transfected with a heavy chain variable region fused to the Fc region of a human IgG (dAb-Fc) [14]. Cells expressing the recombinant protein were selected using 400 µg/mL G418 (Invitrogen) [14]. High expressing cells were enriched using a FACS based surface labelling method which was followed by clonal isolation in a semi-solid medium as described by Song et al. [14]. The producer cell line was cultivated in the same medium as the parental cell line supplemented with 400 µg/mL G418.

### Bioreactor Cultures

**Figure 1:**
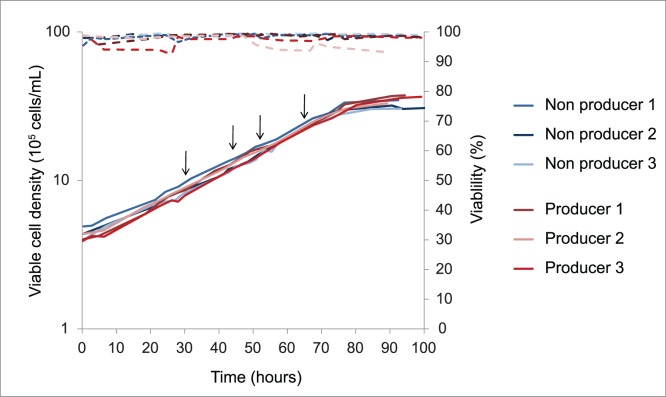
Triplicate growth curves of Hek293 producer and non-producer cell line in batch bioreactor cultures. Arrows indicate sampling time points for metabolomics and transcriptomics.

Parental and recombinant Hek293F cells were cultivated in 1.5 L of Freestyle 293 Expression Medium in a 3 L Applikon bioreactor (Z611000220, Applikon Biotechnologies, The Netherlands). Stirrer speed was 150 rpm, temperature was maintained at 37°C, pH was controlled at 7.0 and pO_2_ at 50% air saturation. Cell number and viability were determined using a CedeX cell counter (Roche Innovatis AG, Bielefeld, Germany). Ammonia concentrations were obtained using a Nova Bioprofiler (Nova Biomedical, Waltham MA, USA). Glucose, lactate and extracellular amino acid concentrations of filtered supernatant samples (0.2 µm, Millipore, North Ryde, Australia) were measured by HPLC as described in Dietmair et al. [15]. Samples for fluxomics were taken at least four times daily. Duplicate samples for transcriptomics and triplicate samples for metabolomics were taken approximately 30, 43, 51 and 67 hours after inoculation of the bioreactor (Figure 1). The oxygen uptake rate (OUR) was determined using the dynamic method [16].

### Transcriptomics Sample Preparation

Microarray samples were processed in compliance with MIAME guidelines and the gene expression data has been deposited in the Gene Expression Omnibus (GEO) database (accession number GSE36094). Approximately 5×10^6^ cells were removed from the bioreactor and centrifuged at 200 rcf for 2 min. The supernatant was discarded and the cell pellet frozen in liquid nitrogen and stored at −80°C. Total RNA was extracted using an RNeasy Midi Kit (75144, Qiagen, Doncaster, Australia) according to the manufacturer’s instructions including on-column digestion of DNA (79254, Qiagen). The purified RNA was stored at −80°C and one sample per time point and fermentation (n = 12 for each cell line) was submitted to the Microarray Facility at the Institute for Molecular Biology at The University of Queensland, Australia for gene expression analysis. The quantity and purity of RNA was determined using a NanoDrop spectrophotometer (Thermo Scientific, Waltham, USA) and an Agilent Bioanalyser 2100 (Agilent Technologies, Santa Clara, USA). The RNA was amplified and labelled using the Illumina TotalPrep RNA Amplification kit (IL1791, Ambion/Applied Biosystems, Austen, USA) according to the manufacturer’s instructions. Hybridisation of labelled cRNA to Illumina HT12v4 chips (Illumina, San Diego, USA), washing of the chips after hybridization and signal detection were performed according to the manufacturer’s instructions. Illumina’s GenomeStudio software was used to convert the scanned images into text files for analysis.

### Transcriptomics Data Analysis

The data from Genome studio was imported into GeneSpring version 11.5.1 (Agilent), log_2_ transformed and quantile normalised without baseline transformation. A filter was applied to remove probes which were not expressed in 12/24 samples (the lower cut-off of the detection p-value was set to >0.99). Significance Analysis of Microarrays (SAM) version 3.11 was applied to identify differentially expressed genes between producer and non-producer cultures [17]. A false discovery rate (FDR) of 5.17% (delta value = 0.7) was applied and all genes with q-values (representing the FDR) less than 0.0517 were considered differentially expressed [18]. The differentially expressed genes were analysed manually and through the use of Ingenuity Pathway Analysis (IPA - Ingenuity® Systems, www.ingenuity.com). IPA canonical pathways and functional analysis identified the pathways and molecular and cellular functions from the IPA library that were most significant to the data set. Fisher’s exact test was used to calculate a p-value determining the probability that the association between the genes in the dataset and the canonical pathway/biological function is explained by chance alone. A p-value <0.05 was considered significant.

### Metabolomics Sample Preparation

Approximately 10 mL of cell suspension (corresponding to 8×10^6^–25×10^6^ cells) was drawn from the bioreactor into a syringe filled with 40 mL of ice cold 0.9% NaCl, transferred to a 50 mL tube and centrifuged at 1000 rcf for 1 min at 0.5°C. The supernatant was discarded and the cell pellet resuspended in 50 mL ice cold 0.9% NaCl for washing. After another centrifugation step, the cell pellet was resuspended in 50% aqueous acetonitrile (Labscan, Gliwice, Poland) and incubated for 10 min at 0°C including several rounds of vortexing. A volume of 1 mL extraction solution was used per 5×10^6^ cells [15]. The extraction solution contained 50 nmol azidothymidine and 250 nmol norvaline per sample which was used as internal standard for HPLC and LC-MS/MS analysis. Metabolite extracts were centrifuged at 30000 rcf for 10 min, the supernatant was transferred to a new 50 ml tube, frozen on dry ice, freeze dried and stored at −80°C until analysis. Prior to analysis, the lyophilized metabolites were resuspended in MilliQ purified water (Millipore, Australia).

### Metabolite Measurements

Nucleotides (NAD, NADP, AMP, GDP, ADP, ATP, CTP, GTP, UTP), UDP-glucuronic acid (UDP-glcA) and amino acids (Asp, Glu, Asn, Ser, Gln, His, Gly, Thr, Arg, Ala, Tyr, Val, Met, Trp, Phe, Ile, Orn, Leu, Lys, Pro) were measured by HPLC as described in Dietmair et al. [15]. TCA cycle (aconitate [ACO], citrate [CIT], isocitrate [ISO], fumarate [FUM], succinate [SUC], malate [MAL], α-ketoglutarate [KGA], oxaloacetate [OAA]) and glycolytic (glucose-6-phophate [G6P], fructose-6-phophate [F6P], fructose-1,6-diphosphate [F16DP], glucose-1-phophate [G1P], glyceraldehy-3-phosphate [GA3P], dihydroxyacetone-phosphate [DHAP], pyruvate [PYR], lactate [LAC], 3-phosphoglycerate/2-phosphoglycerate [3PG/2PG], phosphoenolpyruvate [PEP]) and pentose phosphate pathway (PPP) intermediates (ribose-5-phosphate [R5P], ribulose-5-phosphate [RL5P], xylulose-5-phosphate [X5P]) as well as UDP-glucose (UDP-glc) were measured by LC-MS/MS as described below.

LC-MS/MS data acquisition was performed using a Dionex UltiMate 3000 liquid chromatography system (Dionex, California, USA) coupled to an ABSciex 4000 QTRAP mass spectrometer (ABSciex, Concord, Canada). Chromatographic separation was achieved on a Gemini-NX C18 150×2.0 mm, 3 µm 110 Å particle column (Phenomenex, Aschaffenburg, Germany) maintained at 55°C in the column oven. The mobile phase, adapted from Luo et al. [19], was 7.5 mM aqueous tributylamine solution adjusted to pH 4.95 with glacial acetic acid (eluent A) and acetonitrile (eluent B). The gradient profile is displayed in Table 1. The injection volume was 10 µL. Mobile phase at a flow rate of 0.3 ml/min was introduced directly into the mass spectrometer with no split.

**Table 1:** Gradient profile of LC-MS/MS.

Time	Eluent A (%)
0	100
8	100
20	80
30	73
31	0
33	0
34	100
50	100

The mass spectrometer, equipped with a TurboV electrospray ion source, was operated in negative ionisation mode. The turbo ionspray voltage was set to −4500 V, and the nebuliser (GS1), auxiliary (GS2), curtain (CUR) and collision (CAD) gases were 60, 60, 20 and medium (arbitrary units), respectively, being generated from pressurized air in a N300DR nitrogen generator (Peak Scientific, Massachusetts, USA). The auxiliary gas temperature was maintained at 350°C. Entrance potential was fixed to −10 V for all transitions. Declustering potential, collision energy and collision cell exit potential were dependent upon previously optimized settings for the individual analytes. To obtain adequate selectivity and sensitivity, the mass spectrometer was set to unit resolution and scheduled Multiple Reaction Monitoring mode after determining an updated retention time of each analyte under the chromatographic conditions described above. Sample acquisition order was randomized and two quality control (QC) samples were acquired alternately every 10 sample injections. QC1 was a repeat injection of calibration level 3 (12.5 µM) and QC2 a pooled sample of the entire sample set [20], thus providing assay and sample specific means by which to monitor acquisition stability and reproducibility.

### Metabolomics Data Analysis

HPLC samples were normalised to internal standard (azidothymidine for nucleotides and norvaline for amino acids) and viable cell density. Peak areas of metabolites detected by LC-MS/MS were normalised to the median peak area of that metabolite. In addition, LC-MS/MS samples were normalised to internal standard (azidothymidine) and viable cell number. Normalised HPLC and LC-MS/MS data were uploaded to SIMCA-P v12.0.1 (Umetrics AB, Sweden), mean centered and unit variance scaled. This was followed by multivariate analysis using principal component analysis (PCA) and orthogonal projection to latent structures-discriminant analysis (OPLS-DA). Univariate analysis (ANOVA) was performed using the R statistical software package version 1.6–4 [21].

### Fluxomics

The metabolite consumption and production rates were calculated as described in Quek et al. [22] and used for flux variability analysis (FVA). The biomass composition was obtained from literature values and can be found in Table S1. The cell dry weight of the producer cells was determined in a separate bioreactor culture. Cell suspensions corresponding to at least 3×10^8^ cells were removed at three times during exponential phase and centrifuged at 200 rcf for 5 min. The cell pellets were resuspended in PBS and combined to yield 3×10^8^ cells in 50 mL PBS. The cell suspension was counted three times and centrifuged at 200 rcf for 15 min. The supernatant was carefully removed, the cell pellets frozen on dry ice and lyophilised. The cell dry weight was obtained by subtracting the weight of the empty, pre-dried 50 mL tube from the weight of the same 50 mL tube containing the dried cell pellet.

The metabolic model is a reduced version of the human genome scale model [23]. The model describes 36 biomass drains (amino acid, nucleotides and lipids), a product drain and 347 reactions and transporters required for biosynthesis and energy metabolism distributed across extracellular matrix, cytosol, mitochondria and peroxisomes (Table S2). A total of 60 metabolic rates are measured with two degrees of redundancy enabling gross measurement error analysis and subsequent adjustment of measured rates into a balanceable set for FVA [22]. The model contains one residual degree of freedom representing two alternative pathways to produce NADPH (the cytosolic malic enzyme or oxidative PPP); of 324 calculated fluxes 42 are undetermined, but all are constrained by FVA. For both determined and undetermined fluxes, an approximate 95% confidence interval was calculated using the adjusted rates plus/minus two times the estimated standard errors. The metabolic models are stored in Systems Biology Markup Language (SBML) format, and can be accessed via MATLAB script files available from Prof. Lars K. Nielsen (lars.nielsen@uq.edu.au).

## Results

### Growth Profiles and Nutrient Consumption and Production Rates

The growth profiles of the protein producing HEK293 cell line (producer) and its parental cell line (non-producer) are shown in Figure 1. The exponential growth rates of the two cell lines were the same (0.028±0.001, p = 0.99, ANOVA) and the cell specific productivity of the producer cell line was 13.6±0.1 pg (cell day)^−1^. The coefficients of variation (CV) for growth rates (<4% and <7% for producer and non-producer cells respectively) and cell specific productivity (for the producer cell line, <1%) were small, indicative of stable phenotypes and high reproducibility of the bioreactor cultures. The same mean cell diameter of 15.5±0.3 µm was observed for producer and non-producer (Welch two sample t-test, p = 0.22) and the cell dry weight measured for the producer was assumed representative for both.

Glucose consumption and lactate production in producer cultures were 20% lower than in non-producer cultures (431±38 versus 528±62 µmol (gDW h)^−1^ for glucose consumption and 543±54 versus 695±92 µmol (gDW h)^−1^ for lactate production; Table 2). The molar lactate to glucose yields were similar (1.26±0.17 and 1.31±0.26 for the producer and non-producer respectively) suggesting that glucose utilisation efficiency was not improved in the producer cultures. The amino acid consumption and production pattern was the same for both cell lines. Alanine was the major product, followed by glutamate, glycine, ornithine and proline (Table 2). All other amino acids were consumed. Somewhat surprisingly, serine rather than glutamine was the major non-essential amino acid substrate for the two cell lines. This suggests that Hek293 cells have adapted their metabolism to preferentially use serine, the highest concentrated amino acid in the Freestyle293 medium we employed in this study. Although the error of the glutamine uptake rate estimate was very large (due to the presence of Glutamax, a dipeptide of alanine and glutamine), results suggest that glutamine consumption was lower in the producer cell line (Table 2). Essential amino acid uptake rates were slightly but consistently higher in the producer cell line, consistent with a 3% increase in protein biosynthesis through the recombinant product.

**Table 2:** Mean (n = 3) metabolic uptake and production rates of non-producer and producer cultures during exponential phase.

	Non-producer	Producer	Significantly differentially expressed transporters^1^
Metabolite	Rate ± SE	Rate ± SE	
Glucose*	−528±63	−431±38	GLUT10, GLUT11
Lactate*	695±92	543±54	
Y_lactate/glucose_	1.3±0.2	1.3±0.2	
NH_4_	17±3	17±2	
OUR	−335±29	−334±55	
**Consumed amino acids**			
Gln	−29±17	−6±21	
Asn	−4.1±1.0	−3.9±0.4	
Ser	−37±6	−38±4	SLC1A4
Asp	−6.3±1.6	−7.9±1.1	SLC1A3
**Essential amino acids**			
Arg	−16±3	−17±2	SLC7A6, SLC7A5, SLC3A2
His	−3.2±0.5	−3.4±0.3	
Thr	−8.4±1.6	−8.9±0.9	SLC1A4
Tyr	−4.6±0.7	−4.8±0.4	SLC7A5, SLC3A2
Val	−14±3	−14±2	
Met	−4.9±1.1	−5.0±0.6	SLC7A6
Trp	−1.5±0.3	−1.7±0.2	SLC7A5, SLC3A2
Phe	−5.8±1.0	−5.8±0.6	SLC7A5, SLC3A2
Ile	−13±3	−13±2	
Leu	−19±4	−20±2	SLC7A6, SLC7A5, SLC3A2
Lys	−12±2	−12±1	
**Produced amino acids**			
Ala	11±17	17±24	SLC1A4
Glu	9.6±1.4	8.1±1.1	SLC1A3
Gly	6.2±1.1	6.9±1.1	
Orn	9.0±1.5	7.5±1.5	
Pro	3.8±0.4	3.9±0.7	

Rates are shown in µmol (gDW h)^−1^. Negative rates represent consumption and positive rates production of the corresponding metabolite.

1 Differentially expressed transporters in producer and non-producer cultures according to Significance Analysis of Microarrays (SAM) using a false discovery rate of 5.17%. All amino acid transporters were upregulated in producer cultures. OUR = oxygen uptake rate, SE = standard error, Y = yield,

* indicates rates which were statistically significantly different (p<0.05, ANOVA) in producer and non-producer cultures.

### Fluxomics

**Figure 2:**
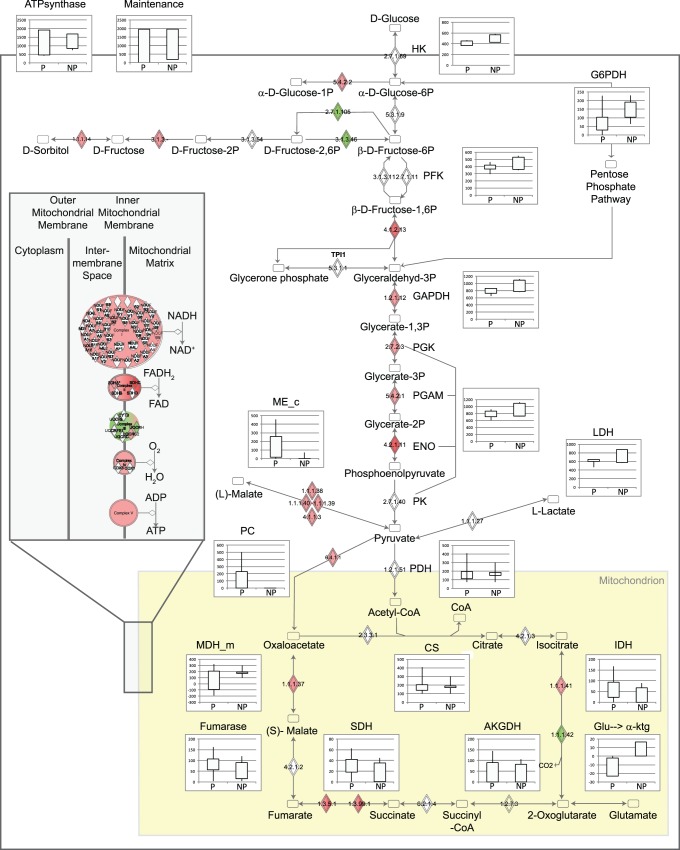
Overlay of fluxomics and transcriptomics results. Intracellular fluxes were calculated using a flux balance approach based on the maximization and minimization of ATP yield and are shown as solid bars with the 95% confidence interval, calculated from the adjusted rates plus/minus two times the estimated standard error, shown as vertical lines. Red/green coloured diamonds indicate which transcripts were down−/upregulated in producer cultures. Figure adapted from Ingenuity Pathway Analysis.

Gross measurement error analysis was performed to confirm that the cell specific consumption and production rates (Table 2) were consistent [22]. The calculated chi-square (χ^2^) test scores were 1.6 and 0.6 for the producer and non-producer data respectively, which were well below the cut-off of 5.99 (χ^2^(2)_95%_) indicating absence of a gross measurement error. The adjusted rates were then used to calculate intracellular metabolic fluxes for producer and non-producer cultures (Figure 2).

Although the majority of flux ranges overlapped, a variety of metabolic differences could be observed between producer and non-producer cultures. As expected from their reduced glucose consumption, producer cultures had a lower glycolytic flux compared to non-producer cultures, indicated by the shift of the glycolytic flux ranges to lower values (e.g., hexokinase [HK], phosphofructokinase [PFK], pyruvate kinase [PK]) (Figure 2). Similar to other Hek293 cells [24], the producer and non-producer cultures converted the majority of pyruvate to lactate (70% and 80% for the producer and non-producer respectively). Non-producer cultures appeared to channel a larger percentage of glucose through the pentose phosphate pathway (PPP) as indicated by the higher glucose-6P-dehydrogenase (G6PDH) flux range in the non-producer. The PPP provides ribose for nucleic acid synthesis as well as NADPH, an important cofactor in a large number of reactions (e.g., fatty acid and nucleotide synthesis). An alternative source for NADPH is the cytoplasmic malic enzyme (ME_c, converting malate to pyruvate), which appeared to be preferred by the producer cell line (Figure 2).

One of the most interesting flux results was the presence of a pyruvate carboxylase (PC) flux in producer but not in non-producer cultures. PC catalyses the conversion of pyruvate to oxaloacetate, representing an alternative route for pyruvate to enter the TCA cycle. However, the majority of TCA cycle fluxes in producer and non-producer cultures appeared to be similar except for the flux through mitochondrial malate dehydrogenase (MDH_m) (Figure 2). The large variability in MDH_m flux of producer cultures is a consequence of the variability in PC flux. As MDH_m and PC both produce mitochondrial oxaloactetate, the presence of a PC flux increases the uncertainties associated with MDH_m flux. Another difference between producer and non-producer cultures was that non-producer cultures converted more glutamine to glutamate and further to α-ketoglutarate, thereby slightly increasing the flux through the lower part of the TCA cycle (Figure 2).

One of the advantages of fluxomics is that it allows calculation of the quantities of ATP produced and consumed in different pathways (Figure 2). Non-producer cultures produced up to 30% more ATP in glycolysis than producer cultures. Despite the reduced quantities of ATP produced in glycolysis, producer cultures achieved the same growth rates as non-producer cultures in addition to producing a recombinant protein. Thus, the producer cultures either had to increase the amount of ATP produced via oxidative phosphorylation, or reduce their overall energy consumption (e.g., by reducing their maintenance energy). While both scenarios are possible according to the calculated fluxes (Figure 2), similar oxygen uptake rates (OUR) for producer and non-producer cultures support the latter (Table 2).

### Metabolomics

**Figure 3:**
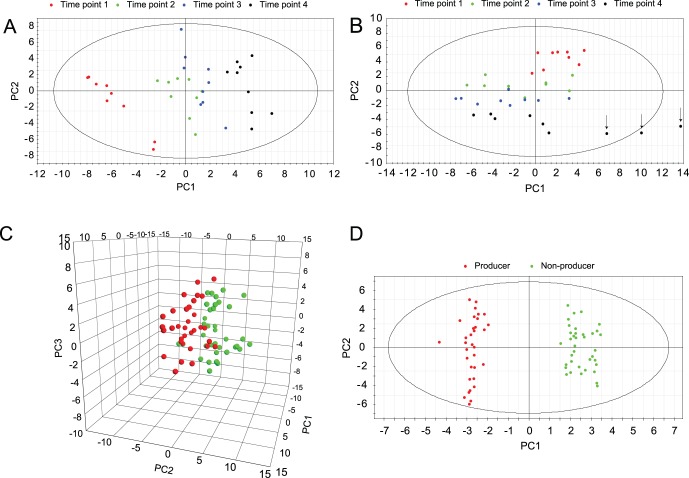
Multivariate analysis of intracellular metabolite concentrations. Principal component analysis (PCA) and orthogonal projection to latent structures - discriminant analysis (OPLS-DA) was used to explore intracellular metabolite concentrations of producer and non-producer cultures. (A) Score scatter plot of the first two principal components of non-producer samples. (B) Score scatter plot of the first two principal components of producer samples. Arrows indicate outliers, believed to be a consequence of incorrect cell counts, which were removed. (C) 3D score scatter plot of the first three principal components of producer (red) and non-producer (green) samples. (D) OPLS-DA score scatter plot of producer (red) and non-producer samples (green).

Samples for intracellular metabolite analysis were taken at four time points during exponential phase in triplicate bioreactor cultures resulting in 36 samples each for the two cell lines (Figure 1). A total of 52 metabolites were measured by HPLC and LC-MS/MS (see Materials and Methods and Table S3). Independent principal component analysis (PCA) of non-producer and producer data was performed to inspect the data (Figure 3A and B). Three outliers, corresponding to producer samples taken at time point 4 of fermentation 3, which were believed to be the consequence of incorrect cell counts, were detected and removed (Figure 3B). A clear time-dependent trend can be observed in both scores plots (Figures 3A and 3B).

PCA of all samples resulted in two slightly separated clusters corresponding to producer and non-producer samples (Figure 3C), which was further delineated by OPLS-DA (Figure 3D). Metabolites that contributed most to the separation of producer and non-producer cultures according to OPLS-DA, with a variable importance/influence on projection (VIP) score of >1, are listed in Table 3.

**Table 3:** Metabolites contributing most to the separation of producer and non-producer cultures according to orthogonal projection to latent structures - discriminant analysis (OPLS-DA) and corresponding fold changes (FC) at time points 1–4.

	Time point 1	Time point 2	Time point 3	Time point 4
Metabolite	FC ± SE	FC ± SE	FC ± SE	FC ± SE
UDP-GlcA	**0.44±0.05**	**0.43±0.04**	**0.41±0.04**	**0.39±0.03**
AMP	**1.70±0.03**	**1.90±0.16**	**1.49±0.20**	**2.14±0.48**
ATP	0.87±0.06	**0.81±0.04**	**0.81±0.03**	**0.82±0.05**
F16DP	**0.43±0.11**	**0.32±0.07**	**0.29±0.04**	**0.59±0.15**
CTP	0.87±0.06	**0.85±0.05**	**0.84±0.02**	**0.81±0.04**
Gln	**0.79±0.05**	**0.69±0.06**	**0.64±0.08**	**0.57±0.10**
GTP	**0.86±0.06**	**0.82±0.05**	**0.86±0.04**	**0.86±0.06**
Arg	0.95±0.03	**0.90±0.03**	**0.85±0.05**	**0.83±0.04**
Pro	0.91±0.06	**0.67±0.05**	**0.78±0.04**	**0.84±0.07**
ADP	0.96±0.05	**0.85±0.05**	**0.81±0.04**	**0.83±0.08**
LAC	**0.89±0.05**	0.91±0.06	**0.86±0.05**	**0.78±0.09**
UTP	0.93±0.05	0.90±0.06	**0.87±0.03**	**0.79±0.05**
Trp	**0.90±0.03**	**0.88±0.04**	**0.85±0.05**	0.91±0.05
Val	0.94±0.04	**0.91±0.04**	**0.86±0.05**	**0.87±0.05**
AEC	0.99±0.01	**0.99±0.00**	1.00±0.00	0.99±0.01
Ile	0.94±0.03	0.93±0.04	**0.88±0.05**	0.92±0.05
Glu	0.94±0.05	0.89±0.06	0.83±0.08	0.87±0.10
Lys	0.98±0.04	**0.94±0.03**	0.90±0.05	**0.86±0.05**
GDP	1.16±0.09	**1.18±0.07**	1.05±0.06	1.09±0.11

Bold text indicates statistically significantly different metabolites (ANOVA, p<0.05). UDP-GlcA = UDP-glucuronic acid, F16DP = fructose-1,6-diphosphate, SE = standard error, n = 9.

Almost half (8/19) of the metabolites identified using OPLS-DA were nucleotides or nucleotide sugars. With the exception of AMP and GDP, all were more abundant in non-producer than in producer cultures (Table 3). Intracellular UDP-glcA and AMP concentrations displayed the greatest differences between producer and non-producer cultures. UDP-glcA concentrations were up to 61% lower while AMP concentrations were up 114% higher in producer cultures. The adenylate energy charge (AEC; defined as [ATP+ADP/2]/[AMP+ADP+ATP] [25]) during exponential phase was slightly higher in non-producer cultures (0.92±0.02 versus 0.93±0.17 for producer and non-producer cultures respectively).

Similar to the majority of nucleotides, the concentration of most glycolytic and TCA cycle intermediates was slightly lower in producer than in non-producer cultures. Except for F16DP, however, these differences were not statistically significant (p>0.05, ANOVA). This lack of significance may reflect, at least in part, a high degree of variability in measurement data obtained by LC-MS/MS (mean CV of 18% for LC-MS/MS versus a mean CV of less than 10% for HPLC measurements). Although not statistically significant, intracellular lactate concentrations were up to 22% lower in producer cells, in which lactate production rates were also lower (Table 2). F16DP concentrations were up to 71% lower in producer cultures. These observations corroborate the calculated flux data for which the glycolytic rate in non-producers was lower than for producers (Figure 2).

Several amino acids (Gln, Arg, Pro, Trp, Val, Ile, Glu, Lys) were identified to significantly contribute to the separation between producer and non-producer cultures using OPLS-DA. The most significant amino acid was Gln, whose concentration was up to 43% lower in producer than in non-producer cultures. This observation may be related to the decreased Gln uptake rate of producers (ca. 5 fold less than the rate for non-producers, Table 2). Intracellular concentrations of Arg, Pro, Trp, Val, Ile, Glu and Lys were also lower in producer than in non-producer cultures, but to a lesser extent than Gln. Interestingly, intracellular Arg and Lys concentrations in non-producer cultures remained relatively constant during exponential phase, while decreasing in producer cultures. The same trend was observed for intracellular Trp, Ile and Val concentrations for time points 1–3 (Table 3). This suggests increased consumption of Arg, Lys, Trp, Ile and Val in producer cultures, which is in agreement with the higher uptake rates of these amino acids.

### Transcriptomics

To investigate differences in gene expression between producer and non-producer cultures, we analysed transcript abundances in both cell lines using an Illumina HT12 chip. Of the 47218 entities present on the chip, 17836 probes were expressed in at least 50% of the samples (12/24). Using an FDR of 5.17%, 2445 probes were differentially regulated between producers and non-producers (Significance Analysis of Microarray - SAM). Of these, 1428 were comparatively down- and 1017 upregulated in the producer cell line (Table S4). Only 72 and 12 probes changed more than 1.5 and 2 fold respectively, highlighting that the magnitude of significant differences between cultures is often small. By applying a fold change (FC) cut-off one might miss relevant genes with small changes in gene expression [26].

The list of differentially regulated probes was uploaded to IPA for further interrogation, and more than 90% could be mapped to the IPA knowledge base. In addition to IPA, visual inspection was performed. The ten genes with the highest positive and negative fold changes according to IPA are listed in Table 4. Only three of these genes immediately appear to be related to protein production, namely ribosomal protein L14 (RPL14), prefoldin subunit 6 (PFDN6) and heat shock 70 kDa protein 6 (Hsp6), which are involved in translation and folding. In contrast to our expectations, however, two of these genes (PFDN6, RPL14) were downregulated in producer cultures. PFDN6 predominantly acts as chaperone for nascent actin and tubulin chains [27]–[29], and hence may not be involved in the folding of the recombinant protein. Notably, the majority of genes encoding actin, tubulin and actin related proteins (e.g., ARPC2, Table 4) were downregulated in producer cells. Two additional genes listed in Table 4 also appear to be related to cytoskeletal organisation: Ras-related C3 botulinum toxin substrate 2 (RAC2) [30]–[33] and kelch-like 4 (KHLH4), an actin binding protein [34]. Together, these observations are suggestive of altered cytoskeletal organisation in producer compared to non-producer cells. However, the relevance of these observations to recombinant protein production is unclear.

**Table 4:** Most significantly regulated genes according to Ingenuity Pathway analysis.

Gene ID	Gene name	Fold change
**Downregulated in producer cultures**		
FABP5	Fatty acid binding protein 5 (psoriasis-associated)	6.31
FABP5L1	Fatty acid binding protein 5 pseudogene 1	5.03
FGGY	FGGY carbohydrate kinase domain containing	4.39
BEX1	Brain expressed, X-linked 1	2.92
SDHC	Succinate dehydrogenase complex, subunit C	2.74
PFDN6	Prefoldin subunit 6	2.64
RPL14	Ribosomal protein L14	2.63
FBXO22	F-box protein 22	2.46
ITM2C	Integral membrane protein 2C	2.28
ARPC2	Actin related protein 2/3 complex, subunit 2	2.22
**Upregulated in producer cultures**		
RETNLB	Resistin like beta	65.5
PDZD2	PDZ domain containing 2	5.90
WDR72	WD repeat domain 72	5.38
ACHE	Acetylcholinesterase	2.35
RYR1	Ryanodine receptor 1 (skeletal)	2.20
RAC2	Ras-related C3 botulinum toxin substrate 2	2.15
KLHL4	Homo sapiens kelch-like 4 (Drosophila)	1.82
RAI14	Retinoic acid induced 14	1.77
HSPA6	Heat shock 70 kDa protein 6	1.75
GOLPH3	Golgi phosphoprotein 3 (coat-protein)	1.66

The two most downregulated genes (Table 4) corresponded to fatty acid binding protein 5 (FABP5) and related sequences (FABP5L1). FABP5 is one of the nine members of the fatty acid binding protein (FABP) family, which regulate lipid uptake, secretion and intracellular transport [35]. Fatty acids fulfil a variety of cellular functions (e.g., energy storage, signalling molecules and transcription factors) but are poorly soluble in the aqueous cytosol requiring FABPs to transport them to their respective destinations [35]. Hence, differential expression of FABPs may affect a variety of cellular functions complicating analysis of their relevance to this study.

**Figure 4:**
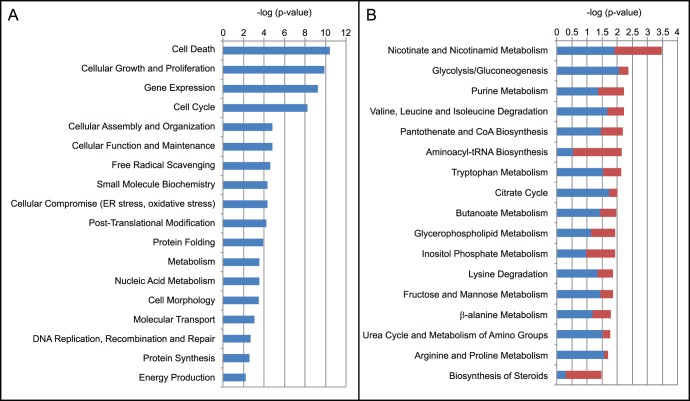
Ingenuity Pathway analysis (IPA) of differentially expressed genes. Relevant and significant (p<0.05, right-tailed Fisher’s exact test) (A) biological functions and (B) metabolic pathways identified using IPA. Red and blue colours represent the percentage of up- and downregulated genes. The function metabolism summarises carbohydrate, lipid, amino acid, vitamin and mineral metabolism. The complete list of functions and pathways can be found in Table S5.

A number of biological functions and canonical pathways were identified by IPA, for which their relevance to the present study is unclear (e.g., germ cell-sertoli cell junction signalling, gastrointestinal disease) and these are not discussed further but can be found in Table S5. Figure 4 gives an overview of relevant biological functions and metabolic pathways identified using IPA (p<0.05, right-tailed Fisher’s exact test). Although no outward difference in growth rate, viability or morphology between producer and non-producer cultures were observed, IPA identified the functional classes cell death, cellular growth and proliferation, cell cycle and cell morphology as being enriched in the list of differentially regulated genes. Notably, approximately 65% of genes belonging to these functions were downregulated in the producer cell line.

### Functions and Pathways Related to Protein Production

IPA identified several biological functions and pathways related to the production of recombinant proteins (e.g., gene expression, protein folding, protein synthesis, aminoacyl-tRNA biosynthesis, Figure 4). One of the most significant functions was gene expression which contained mostly (81%) transcription related genes. While an approximately equal number of transcription related genes were up- and downregulated in producer cultures according to IPA (111 versus 106), inspection of the related canonical pathway purine metabolism revealed that all differentially regulated genes encoding RNA polymerases (POLR2C, POLR2H, POLR3D, POLRMT, POLR1E) were downregulated. In addition, all genes encoding nucleoside diphosphate kinases (NME1, NME2, NME4, NME6) converting NDPs to NTPs (the building blocks of RNA) were also downregulated in producers. Contrary to conventional wisdom, these observations suggest an overall downregulation of transcription in producer cultures.

While translation as such was not identified by IPA, 36% of genes associated with protein synthesis in IPA encoded genes related to translation. Among these were 13 genes encoding translation elongation or initiation factors (EIF3G, EEF1A1, EEF1AL7, LOC402251, LOC649150, EIF3J, EIF2B3, EIF4A2, TSFM, EIF3C/EIF3CL, EIF4G2, EIF2C3, EIF4H), of which only four were upregulated in the producer cell line. In addition, the majority of genes encoding ribosomal proteins were downregulated in producer cultures, suggesting an overall downregulation of translation. In contrast, the majority of genes involved in aminoacyl-tRNA biosynthesis, such as cysteinyl-, lysyl, leucyl, threonyl, tryptophanyl and tyrosyl-tRNA synthetases (CARS, KARS, LARS2, TARS, WARS, YARS) were upregulated in producer cultures (Figure 4).

Overall, an approximately equal number of genes associated with the folding of recombinant proteins were up- and downregulated in the producer cell line suggesting that the protein folding capacity was not substantially increased (Table 5). Although not statistically significant at the 95% level (p = 0.07), the endoplasmic reticulum (ER) stress pathway was also identified by IPA. In contrast to most other pathways which predominantly contained genes that were downregulated in producer cultures, this pathway included four up- and only one downregulated gene (Table 5). Caspase 9 (CASP9), heat shock 70 kDa protein 5 (BiP), homocysteine-inducible, endoplasmic reticulum stress-inducible, ubiquitin-like domain member 1 (HERPUD1) and X-box binding protein 1 (XBP-1) were upregulated while endoplasmic reticulum to nucleus signalling 1 (ERN1) was downregulated.

**Table 5:** Differentially regulated genes involved in protein folding.

Gene ID	Gene name	Fold change	q value (%)
**Protein folding**			
**Upregulated in producer cultures**			
HSPA6	Heat shock 70 kDa protein 6	1.75	0.00
DNAJC21	DnaJ (Hsp40) homolog, subfamily C, member 21	1.57	0.00
HSPA5	Heat shock 70 kDa protein 5 (Grp78, BiP)	1.24	0.00
PDIA4	Protein disulfide isomerase family A, member 4	1.16	2.48
PDIA6	Protein disulfide isomerase family A, member 6	1.15	2.48
HSPA1L	Heat shock 70 kDa protein 1 pseudogene	1.15	1.09
HSP90AA1	Heat shock protein 90 kDa alpha, class A member 1	1.13	2.48
CANX	Calnexin	1.11	2.48
FKBP1A	FK506 binding protein 1A	1.11	1.09
**Downregulated in producer cultures**			
DNAJC11	DnaJ (Hsp40) homolog, subfamily C, member 11	1.53	0.00
HSP 84	Similar to Heat shock protein HSP 90-beta	1.38	0.00
PDIA5	Protein disulfide isomerase family A, member 5	1.16	0.00
CALR	Calreticulin	1.16	0.10
HSP90AB4P	Heat shock protein 90 kDa alpha, class B member 4, pseudogene	1.16	2.48
HSP90AB1	Heat shock protein 90 kDa alpha, class B member 1	1.16	0.53
PPIA	Peptidylprolyl isomerase A	1.14	2.48
HSPA2	Heat shock 70 kDa protein 2	1.14	0.53
PDIA3P	Protein disulfide isomerase family A, member 3, pseudogene	1.13	2.48
ERP44	ER protein 44	1.12	2.48
**ER stress**			
**Upregulated in producer cultures**			
XBP1	X-box binding protein 1	1.28	0.00
HERPUD1	Homocysteine-inducible, ER stress-inducible, ubiquitin-like domain member 1	1.26	0.00
HSPA5	Heat shock 70 kDa protein 5 (Grp78, BiP)	1.24	0.00
CASP9	Caspase 9	1.12	1.09
**Downregulated in producer cultures**			
ERN1	Endoplasmic reticulum to nucleus signalling 1	1.15	0.53

The genes listed in this table were identified using Ingenuity Pathway Analysis (IPA) and through visual inspection of the list of differentially expressed genes identified using Significance Analysis for Microarrays (SAM, q <5.17%). Genes involved in the folding of specific proteins (e.g., actin), and hence apparently unrelated to folding of the recombinant protein, were removed.

### Differential Regulation of Metabolic Genes

In addition to production of a recombinant protein, producer cultures were metabolically different. As such, it was not surprising that IPA identified a range of metabolic pathways as differentially regulated between producer and non-producer cultures (e.g., glycolysis, citrate cycle) (Figure 4B). The most significant pathway was nicotinate and nicotinamide metabolism suggesting differences in NADP and NAD usage between producer and non-producer cultures as indicated by the differences in PPP and Me_c fluxes calculated using FBA (Figure 2).

The second most significant pathway was glycolysis, with 14 differentially regulated enzymes/isoenzymes: acylphosphatase (ACYP2), aldehyde dehydrogenase (ALDH1A2, ALDH1A3, ALDH1B1, ALDH3A2), aldolase (ALDOA), glyceraldehyd-3P-dehydrogenase (GAPDH), phosphoglycerate kinase 1 (PGK1), phosphoglycerate mutase (PGAM1, PGAM4), enolase (ENO2) and phosphoglucomutase (PGM1, PGM2, PGM5) (Table 6). With exception of ACYP2 and ALDH1A3, transcripts for these enzymes/isoenzymes were downregulated in producer cells (Table 6).

**Table 6:** Differentially regulated metabolic genes involved in glycolysis, the citrate cycle and oxidative phosphorylation.

Gene ID	Gene name	Fold change	q value (%)
**Glycolysis/gluconeogenesis**			
ACYP2	Acylphosphatase 2, muscle type	1.19	0.10
ALDH1A2	Aldehyde dehydrogenase 1 family, member A2	−1.57	0.00
ALDH1A3	Aldehyde dehydrogenase 1 family, member A3	1.15	1.09
ALDH1B1	Aldehyde dehydrogenase 1 family, member B1	−1.15	0.15
ALDH3A2	Aldehyde dehydrogenase 3 family, member A2	−1.15	1.09
ALDOA	Aldolase A, fructose-bisphosphate	−1.65	0.00
ENO2	Enolase 2 (gamma, neuronal)	−1.84	0.00
GAPDH	Glyceraldehyde-3-phosphate dehydrogenase	−1.16	0.10
PGAM1	Phosphoglycerate mutase 1 (brain)	−1.15	0.53
PGAM4	Phosphoglycerate mutase family member 4	−1.26	0.00
PGK1	Phosphoglycerate kinase 1	−1.10	2.48
PGM1	Phosphoglucomutase 1	−1.31	0.00
PGM2	Phosphoglucomutase 2	−1.14	0.53
PGM5	Phosphoglucomutase 5	−1.27	0.00
**Citrate cycle**			
IDH1	Isocitrate dehydrogenase 1 (NADP+), soluble	−1.15	0.15
IDH2	Isocitrate dehydrogenase 2 (NADP+), mitochondrial	1.17	1.09
IDH3B	Isocitrate dehydrogenase 3 (NAD+) beta	−1.12	0.53
MDH2	Malate dehydrogenase 2, NAD (mitochondrial)	−1.24	0.00
PC	Pyruvate carboxylase	−1.15	0.15
SDHA	Succinate dehydrogenase complex, subunit A	−1.22	0.15
SDHC	Succinate dehydrogenase complex, subunit C	−2.74	0.00
**Oxidative phosphorylation**			
ATP5C1	ATP synthase	−1.16	0.53
ATP5J2	ATP synthase	−1.09	2.48
ATP5L	ATP synthase	−1.12	2.48
**ATP5S**	**ATP synthase**	−**1.19**	**0.10**
**ATPAF1**	**ATP synthase**	−**1.14**	**0.10**
COX8A	Cytochrome c oxidase subunit VIIIA (ubiquitous)	−1.12	1.09
NDUFA10	NADH dehydrogenase 1 alpha subcomplex 10	−1.16	0.15
NDUFB2	NADH dehydrogenase 1 beta subcomplex 2	−1.24	0.00
NDUFB9	NADH dehydrogenase 1 beta subcomplex 9	−1.18	0.00
UQCRC2	Ubiquinol-cytochrome c reductase core protein II	−1.22	2.48
UQCRH	Ubiquinol-cytochrome c reductase hinge protein	−2.74	0.53

Genes in this table were identified using Ingenuity Pathway analysis (IPA) and through visual inspection of the list of differentially expressed genes determined using Significance Analysis for Microarrays (SAM, q<5.17%, bold text). A positive/negative fold change indicates up/downregulation in producer cultures.

In addition to glycolytic enzymes, a large number of genes encoding TCA cycle enzymes/isoenzymes and proteins involved in oxidative phosphorylation were differentially regulated in producer cultures (Table 6). Examples include isocitrate dehydrogenase (IDH1, IDH2, IDH3), succinate dehydrogenase (SDHA, SDHC), malate dehydrogenase (MDH2), NADH dehydrogenase (NDUFA10, NDUFB2, NDUFB9), ubiquinol cytochrome c oxidoreductase (UQCRC2, UQCRH), cytochrome c oxidase (COX8A) and several ATP synthases (ATP5C1, ATP5J2, ATP5L, ATP5S, ATPAF1). With the exception of IDH2 and UQCRH, all of these genes were downregulated in producer cultures (Table 6). If transcript abundance is indicative of the magnitude of flux (e.g., in the absence of other regulatory mechanisms), downregulation of these genes suggests that producer cultures had lower TCA cycle and ATP synthase fluxes compared to non-producer cultures. While this is possible according to the flux results, one cannot draw a definite conclusion due to the large overlap of calculated TCA cycle and ATP synthase fluxes between producer and non-producer cultures (Figure 2).

## Discussion

This study explored metabolic differences between an efficient Hek293 producer line and the parent non-producer, in order to guide future design of superior cell lines for stable and transient recombinant protein production. While not as high as seen in CHO production lines, a specific productivity of 13.6±0.1 pg (cell day)^−1^ is excellent for Hek293 cells, particularly considering that the cells were not subjected to gene amplification. Importantly, no outward phenotypic differences in growth, viability or morphology were apparent between the producer and parent line (Figure 1). Hereby, we avoid the confounding effects of reduced growth rate and its broad cellular consequences, which may obscure changes related to productivity [36], [37].

Against this common phenotypic background, we identified a number of differentially regulated genes associated with the functional classes of cell growth and proliferation, cell cycle, cell death, and cell morphology. While this may simply be reflective of the inherent biological variability between a parental line and its derivate, it may also reflect as yet unknown roles for these functions related to protein production.

Alternatively, as the majority of these genes were downregulated in the producer cell line, the overrepresentation of these functions may reflect a mechanism whereby cells free up resources required for the production of the recombinant protein, while maintaining the same growth rate. It is interesting that although glucose consumption and glycolytic ATP production in producer cultures was lower (Table 2 and Figure 2), there is no metabolic evidence of compensation (e.g., increased Gln or oxygen consumption, increased efficiency of glucose utilisation) based on our measurements. In contrast, many genes encoding TCA cycle enzymes and proteins involved in oxidative phosphorylation were downregulated in producer cultures indicating lower ATP production via oxidative phosphorylation in these cells (Table 6).

### Investigation of Metabolic Differences between Producer and Non-producer Cultures

Identification of the mechanisms underlying reduced glucose uptake and lactate production is of great interest to the pharmaceutical industry, as reduced lactate concentrations generally result in increased viable cell densities and higher product titers [38]–[40]. In our study, producer cultures exhibited a 1.2 fold reduction in glucose uptake and a 1.3 fold reduction in lactate production rates compared to non-producer cultures.

Several studies have shown a positive correlation between the mRNA abundance of the glucose transporter GLUT1 and glucose uptake rate. For example, Flier et al. [41] observed that increased glucose uptake of fibroblasts transfected with ras and src oncogenes correlated with increased expression of GLUT1. Similarly, Paredes et al. [42] showed that knockdown of the glucose transporter GLUT1 decreased glucose uptake of hybridoma cells by 45%. Therefore, we compared the expression levels of GLUT1 and other glucose transporters in producer and non-producer cultures. A total of eight glucose transporters were expressed, namely GLUT1, GLUT3, GLUT6, GLUT8, GLUT10, GLUT11 and GLUT12, of which GLUT1 and GLUT3 had the highest expression levels. In contrast to our expectations, the transcript levels of the majority of glucose transporters were slightly higher in producer than in non-producer cultures, despite their reduced glucose uptake rate. However, only the expression of GLUT10 and GLUT11 was significantly higher in producer cultures (SAM, q<5.17%). This suggests that the reduced glucose uptake rate was not mediated by reduced expression of glucose transporters.

The reduction in glucose consumption of producer cultures led to an approximately 1.2 fold reduction in calculated glycolytic flux (Figure 2). While none of the rate-limiting enzymes (HK, PFK, PK) were differentially expressed between producer and non-producer cultures, a large number of other glycolytic genes were downregulated between 1.1 and 1.9 fold in producer cultures (Table 6). This suggests that the reduction in glycolytic flux was - at least in part - regulated at the transcript level. Thus, it appears that lower glucose uptake may be a consequence of downstream regulation, as opposed to regulation of transporter expression. Alternatively, the reduced expression of glycolytic genes in producer cultures may be a response to lower glycolytic requirements.

**Figure 5:**
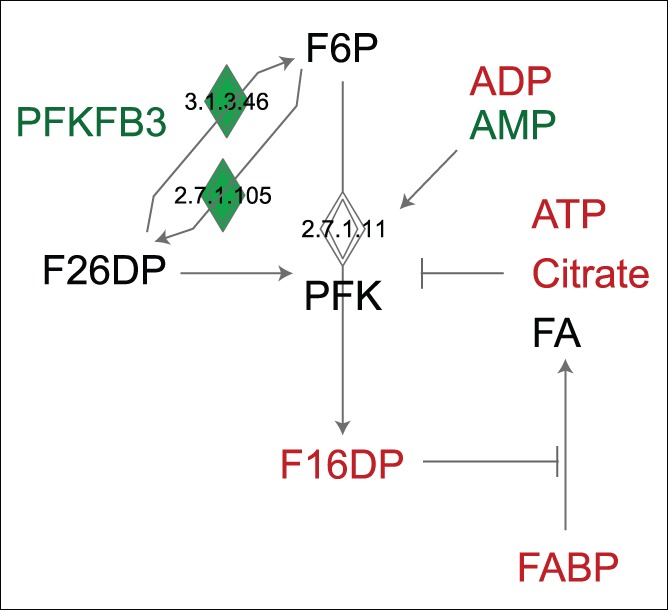
Regulation of phosphofructokinase (PFK). PFK converts fructose-6-phosphate (F6P) to fructose-1,6-diphosphate (F16DP), can be inhibited by ATP, citrate and fatty acids (FA) and activated by ADP, AMP and fructose-2,6-diphosphate (F26DP). F26DP concentrations depend on 6-phosphofructo-2-kinase/fructose-2,6-biphosphatase 3 (PFKFB3) activity converting F6P to F26DP and vice versa. In addition, intracellular FA concentrations depend on fatty acid binding proteins (FABP) which mediate their uptake and intracellular transport. Red and green colours indicate decreased/increased abundance of metabolites/mRNA in producer cultures.

While PFK was not differentially expressed, the concentration of F16DP (a product of PFK), was significantly lower in producer cultures (approximately half, p<0.05, ANOVA, Table 3). While not statistically significant (p>0.05, ANOVA), F6P, the substrate of PFK, was higher in producer than in non-producer cultures at three of the four time points (Table S3), indicating that PFK activity was regulated by other means. Regulation of PFK is complex, and some factors influencing its activity are shown in Figure 5. PFK is activated by AMP, ADP and fructose-2,6-diphosphate (F26DP), and inhibited by ATP, citrate and fatty acids (FA) [43]. As mentioned earlier, FA are poorly soluble in the aqueous cytosol and their intracellular concentration depends on the concentration of FABPs [44]. Interaction of FABPs with FA can be inhibited by F16DP [44]. Several of our observations (higher AMP, lower ATP and citrate concentrations) support higher PFK activity in producer cultures while others (lower ADP and F16DP concentrations, reduced expression of FABP5) support lower PFK activity. Further observations such as an 1.1 fold higher expression of 6-phosphofructo-2-kinase/fructose-2,6-biphosphatase 3 (PFKFB3), converting F6P to F26DP and vice versa, add to the level of complexity. Consequently, the reason for lower F16DP concentrations in producer cultures remains unclear.

Regulation of PFK is one of several examples linking glucose metabolism to FAs and FABPs [44], [45]. FABPs in general have been heavily scrutinised for their role in cellular glucose uptake and insulin resistance with respect to diabetes (reviewed in [35]). Employing a cell culture setting, Kusudo et al. [46] observed that FABP3 expression increased the glucose uptake rate of C2C12 mouse skeletal muscle cells. In the present case, we observed that FABP5 expression was downregulated up to 6.3 fold in producer cells (Table 4), and, while speculative, this may be associated with the reduction in glucose uptake rate. Downregulation of FABP5 (and FABP4) was also observed in hybridoma cells with reduced glucose uptake rates [38], [47]. A 66 fold upregulation of resistin like β (RETNLB) might also be related to the altered glucose metabolism in producer cells (Table 4). RETNLB belongs to a family of cysteine rich secreted proteins composed of resistin, resistin like α and RETNLB [48]. Similar to FABPs, these proteins (especially resistin) have been investigated for their role in insulin resistance and diabetes [49]–[52]. Moon et al. [53] showed that the expression of resistin in rat skeletal muscle cells resulted in a reduction of their glucose uptake rates. In contrast to resistin, the role of RETNLB in humans has hardly been investigated [54], and its association with the reduced glucose uptake rate of producer cultures is merely speculative.

In contrast to glucose uptake rate which did not positively correlate with the expression level of glucose transporters, we observed both increased uptake rates of many amino acids and increased expression of several amino acid transporters (SLC1A4, SLC1A3, SLC7A6, SLC7A5, SLC3A2) in producer cultures (Table 1). Despite higher amino acid uptake, and downregulation of valine leucine and isoleucine degradation pathways (Figure 4), intracellular amino acid concentrations (with the exception of His, Thr, Ser and Asp) were lower in producer cultures. This may relate to higher amino acid utilisation for the purposes of recombinant protein production. Similarly, increased demand of cysteinyl-tRNA synthetase for the cysteine-rich recombinant protein and RETNLB was reflected in increased mRNA abundance.

Our flux analysis indicates that pyruvate carboxylase (PC) was active in the producer but not in the non-producer cells (Figure 2). Hek293 cells deliberately engineered to express yeast pyruvate carboxylase 2 (PYC2) displayed metabolic changes similar to those observed in producer cultures [40]. PYC2 expressing Hek293 cells exhibited lower glucose and glutamine consumption, and a reduced PPP flux (7% versus 28% of glucose uptake rates) than parental cells [40]. Reduced glucose consumption and lactate production has also been observed in CHO cells expressing human PC [55] and BHK cells expressing yeast PC [56], [57]. The similarity suggests that increased PC activity might explain the metabolic differences, though there are several caveats. Firstly, PC has been overexpressed in the cytosol, while the native enzyme is mitochondrial. Secondly, PC expression was slightly lower (1.15 fold) in producer than in non-producer cultures (Table 6) and intracellular malate, pyruvate and oxaloacetate concentrations were similar (Table S3). Thus, neither increased enzyme capacity nor increased thermodynamic driving force can explain the increased flux. It is possible, however, that a change in allosteric regulation (PC is normally activated by acetyl CoA and inhibited by aspartate) can have resulted in the increased activity and downstream effects.

### Characterising Protein Production

The production of a recombinant protein by a mammalian cell, directly affects many cellular processes such as transcription, translation and protein folding. Therefore it is not surprising that many omics studies investigating cell lines with different productivities, have shown differential regulation of genes and proteins involved in these functions [58]–[64]. Similarly, the functions gene expression (containing mostly transcription related genes), post-translational modification, protein folding and synthesis, were overrepresented among differentially expressed genes in our producer cell line (Figure 4). In contrast to our expectations however, we did not observe an overall upregulation of these processes. In fact, many genes related to transcription and translation were downregulated in producer compared to non-producer cultures (e.g., RNA polymerases, nucleoside diphosphate kinases, translation initiation and elongation factors). Potentially, this has a negative impact on recombinant protein production by limiting the rate of transcription and/or translation. Alternatively, the downregulation of these genes may be a consequence of broader adaptive changes not specifically related to protein production. For example, all genes encoding histones (HIST2H2AA3, HIST2H2AC, HIST2H4A, HIST2H2AA4, H2AFY2, H2AFY, HIST3H2A, HIST1H4C, H1F0, H1FX), except for LOC644914, were upregulated in the producer cell line, suggesting increased packing of DNA and therefore reduced accessibility by transcriptional machinery.

Several omics studies have shown that genes and proteins involved in protein folding were upregulated in high producers, indicating that protein folding may pose a bottleneck in certain cell lines [59]–[61], [63], [65]. While some of the genes/proteins identified in these studies were also upregulated in our study (e.g., BiP, PDI, Canx), overall, an approximately equal number of genes involved in protein folding were up- and downregulated. We did however, observe upregulation of several genes related to ER stress in producer cultures, including XBP-1, BiP and HERPUD1 (Table 5). ER stress can be caused by the accumulation of unfolded proteins, leading to activation of the unfolded protein response (UPR) [66]. In its spliced form, XBP-1 is one of the key regulators of the UPR, initiating the transcription of a range of other UPR genes such as BiP, DNAJC21 and FKBP1A [67], [68]. All of these genes were upregulated in producer cultures, suggesting that the UPR was activated and that protein folding capacity may limit recombinant protein production in this cell line. Therefore, overexpression of genes involved in protein folding may help to increase the productivity of producer cultures.

### Conclusions

Hek293 cells are the predominant host for transient expression of recombinant proteins and are also used as hosts for stable expression of proteins where glycosylation is inadequate in other cells. In contrast to CHO cells which have been extensively studied, very few studies have investigated cellular features required for recombinant protein production in Hek293 cells. To shed light on the cellular changes associated with high productivity in these cells, we compared a high producing Hek293 cell line and its parental cell line using transcriptomics, metabolomics and fluxomics.

In many instances, a positive correlation between changes in transcript abundance, fluxes and metabolite concentrations were observed. Perhaps of greater interest are several instances where discrepancies between transcript abundances and fluxes were identified, and/or outcomes were contrary to expectations (e.g., increased expression of glucose transporter genes yet lower glycolytic flux for producer cells). These instances are indicative of alternative regulatory mechanisms which may warrant further investigation.

Overall, we speculate that for the cell lines under investigation, broad adaptation through many subtle changes results in a rebalancing of metabolism and freeing up of metabolic resources required for the production of recombinant protein, leading to a possible bottleneck at the point of protein folding and assembly.

Our integrated omics approach to characterising cellular phenotype identifies features and raises questions that single omics techniques in isolation would have failed to identify. Thus, we illustrate a platform for rational and insightful hypothesis generation in investigating the complexities of cellular phenotype, and developing a deeper understanding of the mechanisms of recombinant protein production. The addition of further technologies such as proteomics and use of next-generation sequencing technologies will undoubtedly enhance the power of this approach, shedding further light on the subtleties of the cellular machine.

## Supporting Information

Table S1 **Biomass composition of Hek293 cells.** Relative percentages of individual metabolites were taken from the literature and multiplied with the measured cell dry weight (514 pg/cell). The relative percentages of total carbohydrates, proteins, lipids, DNA and RNA were taken from Zupke and Stephanopoulos [1] and Bonarius et al. [2]. The amino acid composition of proteins was taken from Okayasu et al. [3] and Sheik et al. [4]. The relative percentages of individual phospholipids, dNTPs and NTP was taken from Sheik et al. [4]. The relative percentages were slightly adjusted to yield 100%.
(DOCX)

Table S2 **Metabolic model of Hek293 cells.**
(DOCX)

Table S3 **Mean intracellular metabolite concentrations in producer and non-producer cultures at time points 1–4.** HPLC measurements are in fmol/cell. LC-MS/MS measurements are relative concentrations based on normalized peak areas. N = 9 except for time point 4 in producer cultures where n = 6. SE = standard error.
(XLSX)

Table S4 **List of differentially regulated genes identified using Significance Analysis of Microarrays (SAM) using a delta value of 0.7 and a false discovery rate (FDR) of 5.17.** A positive fold change (red text) means that the corresponding gene was more abundant in non-producer cultures. A negative fold change (green text) means that the gene was more abundant in producer cultures.
(XLSX)

Table S5 **Complete list of biofunctions and canonical pathways identified using Ingenuity pathway analysis (IPA).**
(XLSX)
