# Supplementary material for: A Multi-Omics Analysis of Recombinant Protein Production in Hek293 Cells
Source: PLoS One. 2012 Aug 24;7(8):e43394. doi: 10.1371/journal.pone.0043394 (PMC3427347; doi:10.1371/journal.pone.0043394)
Supplement: Table S1 — Biomass composition of Hek293 cells. Relative percentages of individual metabolites were taken from the literature and multiplied with the measured cell dry weight (514 pg/cell). The relative percentages of total carbohydrates, proteins, lipids, DNA and RNA were taken from Zupke and Stephanopoulos [1] and Bonarius et al. [2]. The amino acid composition of proteins was taken from Okayasu et al. [3] and Sheik et al. [4]. The relative percentages of individual phospholipids, dNTPs and NTP was taken from Sheik et al. [4]. The relative percentages were slightly adjusted to yield 100%. (DOCX) [file pone.0043394.s001.docx]

| **Metabolite** | **pg/cell** | **Metabolite** | **pg/cell** |
| --- | --- | --- | --- |
| Ala | 32.1 | Cholesterol | 25.8 |
| Arg | 20.2 | Sphingolipids | 15.5 |
| Asn | 15.4 | Phosphatidylcholine | 27.6 |
| Asp | 19.2 | Phosphatidylethanolamine | 10.4 |
| Cys | 7.8 | Phosphatidylinositol | 4.0 |
| Gln | 17.2 | Phosphatidylserine | 1.2 |
| Glu | 20.6 | Phosphatidylglycerol | 0.40 |
| Gly | 28.8 | Diphosphatidylglycerol | 1.20 |
| His | 7.6 | **SUM Lipids** | **86.0** |
| Ile | 17.3 | AMP | 4.64 |
| leu | 30.1 | CMP | 7.74 |
| Lys | 30.5 | GMP | 7.74 |
| Met | 7.4 | UMP | 4.64 |
| Phe | 11.7 | **SUM RNA** | **24.8** |
| Pro | 16.7 | dAMP | 2.01 |
| Ser | 23.0 | dCMP | 1.34 |
| Thr | 20.6 | dGMP | 1.34 |
| Trp | 2.4 | dTMP | 2.01 |
| Tyr | 9.7 | **SUM DNA** | **6.70** |
| Val | 22.2 | **Carbohydrates** | **36.0** |
| **SUM proteins** | **360.5** | **Cell dry weight** | **514** |
