# Supplementary material for: A Multi-Omics Analysis of Recombinant Protein Production in Hek293 Cells
Source: PLoS One. 2012 Aug 24;7(8):e43394. doi: 10.1371/journal.pone.0043394 (PMC3427347; doi:10.1371/journal.pone.0043394)
Supplement: Table S2 — Metabolic model of Hek293 cells. (DOCX) [file pone.0043394.s002.docx]

| **#** | **b** | **c** | **d** |
| --- | --- | --- | --- |
| **1** | ENO | 1 _2pg_c = 1 h2o_c + 1 pep_c | 1 D_Glycerate2_phosphate = 1 H2O + 1 Phosphoenolpyruvate |
| **2** | FBA | 1 fdp_c = 1 dhap_c + 1 g3p_c | 1 D_Fructose1_6_bisphosphate = 1 Dihydroxyacetonephosphate + 1 Glyceraldehyde3_phosphate |
| **3** | HEX1 | 1 atp_c + 1 glc_D_c = 1 adp_c + 1 g6p_c + 1 h_c | 1 ATP + 1 D_Glucose = 1 ADP + 1 D_Glucose6_phosphate + 1 H+ |
| **4** | GAPD | 1 g3p_c + 1 nad_c + 1 pi_c = 1 _13dpg_c + 1 h_c + 1 nadh_c | 1 Glyceraldehyde3_phosphate + 1 Nicotinamideadeninedinucleotide + 1 Phosphate = 1 _3_Phospho_D_glyceroylphosphate + 1 H+ + 1 Nicotinamideadeninedinucleotide_reduced |
| **5** | PYK | 1 adp_c + 1 h_c + 1 pep_c = 1 atp_c + 1 pyr_c | 1 ADP + 1 H+ + 1 Phosphoenolpyruvate = 1 ATP + 1 Pyruvate |
| **6** | PFK | 1 atp_c + 1 f6p_c = 1 adp_c + 1 fdp_c + 1 h_c | 1 ATP + 1 D_Fructose6_phosphate = 1 ADP + 1 D_Fructose1_6_bisphosphate + 1 H+ |
| **7** | PGI | 1 g6p_c = 1 f6p_c | 1 D_Glucose6_phosphate = 1 D_Fructose6_phosphate |
| **8** | PGM | 1 _2pg_c = 1 _3pg_c | 1 D_Glycerate2_phosphate = 1 _3_Phospho_D_glycerate |
| **9** | PGK | 1 _3pg_c + 1 atp_c = 1 _13dpg_c + 1 adp_c | 1 _3_Phospho_D_glycerate + 1 ATP = 1 _3_Phospho_D_glyceroylphosphate + 1 ADP |
| **10** | TPI | 1 dhap_c = 1 g3p_c | 1 Dihydroxyacetonephosphate = 1 Glyceraldehyde3_phosphate |
| **11** | GND | 1 _6pgc_c + 1 nadp_c = 1 co2_c + 1 nadph_c + 1 ru5p_D_c | 1 _6_Phospho_D_gluconate + 1 Nicotinamideadeninedinucleotidephosphate = 1 CO2 + 1 Nicotinamideadeninedinucleotidephosphate_reduced + 1 D_Ribulose5_phosphate |
| **12** | PGL | 1 _6pgl_c + 1 h2o_c = 1 _6pgc_c + 1 h_c | 1 _6_phospho_D_glucono_1_5_lactone + 1 H2O = 1 _6_Phospho_D_gluconate + 1 H+ |
| **13** | RPI | 1 r5p_c = 1 ru5p_D_c | 1 alpha_D_Ribose5_phosphate = 1 D_Ribulose5_phosphate |
| **14** | RPE | 1 ru5p_D_c = 1 xu5p_D_c | 1 D_Ribulose5_phosphate = 1 D_Xylulose5_phosphate |
| **15** | TALA | 1 g3p_c + 1 s7p_c = 1 e4p_c + 1 f6p_c | 1 Glyceraldehyde3_phosphate + 1 Sedoheptulose7_phosphate = 1 D_Erythrose4_phosphate + 1 D_Fructose6_phosphate |
| **16** | TKT1 | 1 r5p_c + 1 xu5p_D_c = 1 g3p_c + 1 s7p_c | 1 alpha_D_Ribose5_phosphate + 1 D_Xylulose5_phosphate = 1 Glyceraldehyde3_phosphate + 1 Sedoheptulose7_phosphate |
| **17** | TKT2 | 1 e4p_c + 1 xu5p_D_c = 1 f6p_c + 1 g3p_c | 1 D_Erythrose4_phosphate + 1 D_Xylulose5_phosphate = 1 D_Fructose6_phosphate + 1 Glyceraldehyde3_phosphate |
| **18** | FDH | 1 for_c + 1 nad_c = 1 co2_c + 1 nadh_c | 1 Formate + 1 Nicotinamideadeninedinucleotide = 1 CO2 + 1 Nicotinamideadeninedinucleotide_reduced |
| **19** | LDH_L | 1 lac_L_c + 1 nad_c = 1 h_c + 1 nadh_c + 1 pyr_c | 1 L_Lactate + 1 Nicotinamideadeninedinucleotide = 1 H+ + 1 Nicotinamideadeninedinucleotide_reduced + 1 Pyruvate |
| **20** | PPA | 1 h2o_c + 1 ppi_c = 1 h_c + 2 pi_c | 1 H2O + 1 Diphosphate = 1 H+ + 2 Phosphate |
| **21** | ME2 | 1 mal_L_c + 1 nadp_c = 1 co2_c + 1 nadph_c + 1 pyr_c | 1 L_Malate + 1 Nicotinamideadeninedinucleotidephosphate = 1 CO2 + 1 Nicotinamideadeninedinucleotidephosphate_reduced + 1 Pyruvate |
| **22** | TRDR | 1 h_c + 1 nadph_c + 1 trdox_c = 1 nadp_c + 1 trdrd_c | 1 H+ + 1 Nicotinamideadeninedinucleotidephosphate_reduced + 1 Oxidizedthioredoxin = 1 Nicotinamideadeninedinucleotidephosphate + 1 Reducedthioredoxin |
| **23** | G3PD1 | 1 glyc3p_c + 1 nad_c = 1 dhap_c + 1 h_c + 1 nadh_c | 1 Glycerol3_phosphate + 1 Nicotinamideadeninedinucleotide = 1 Dihydroxyacetonephosphate + 1 H+ + 1 Nicotinamideadeninedinucleotide_reduced |
| **24** | ASNS1 | 1 asp_L_c + 1 atp_c + 1 gln_L_c + 1 h2o_c = 1 amp_c + 1 asn_L_c + 1 glu_L_c + 1 h_c + 1 ppi_c | 1 L_Aspartate + 1 ATP + 1 L_Glutamine + 1 H2O = 1 AMP + 1 L_Asparagine + 1 L_Glutamate + 1 H+ + 1 Diphosphate |
| **25** | ASPTA | 1 akg_c + 1 asp_L_c = 1 glu_L_c + 1 oaa_c | 1 _2_Oxoglutarate + 1 L_Aspartate = 1 L_Glutamate + 1 Oxaloacetate |
| **26** | ALATA_L | 1 akg_c + 1 ala_L_c = 1 glu_L_c + 1 pyr_c | 1 _2_Oxoglutarate + 1 L_Alanine = 1 L_Glutamate + 1 Pyruvate |
| **27** | CBPS | 2 atp_c + 1 gln_L_c + 1 h2o_c + 1 hco3_c = 2 adp_c + 1 cbp_c + 1 glu_L_c + 2 h_c + 1 pi_c | 2 ATP + 1 L_Glutamine + 1 H2O + 1 Bicarbonate = 2 ADP + 1 Carbamoylphosphate + 1 L_Glutamate + 2 H+ + 1 Phosphate |
| **28** | TYRTA | 1 akg_c + 1 tyr_L_c = 1 _34hpp_c + 1 glu_L_c | 1 _2_Oxoglutarate + 1 L_Tyrosine = 1 _3__4_Hydroxyphenylpyruvate + 1 L_Glutamate |
| **29** | PRPPS | 1 atp_c + 1 r5p_c = 1 amp_c + 1 h_c + 1 prpp_c | 1 ATP + 1 alpha_D_Ribose5_phosphate = 1 AMP + 1 H+ + 1 _5_Phospho_alpha_D_ribose1_diphosphate |
| **30** | PGCD | 1 _3pg_c + 1 nad_c = 1 _3php_c + 1 h_c + 1 nadh_c | 1 _3_Phospho_D_glycerate + 1 Nicotinamideadeninedinucleotide = 1 _3_Phosphohydroxypyruvate + 1 H+ + 1 Nicotinamideadeninedinucleotide_reduced |
| **31** | PSP_L | 1 h2o_c + 1 pser_L_c = 1 pi_c + 1 ser_L_c | 1 H2O + 1 O_Phospho_L_serine = 1 Phosphate + 1 L_Serine |
| **32** | AHC | 1 ahcys_c + 1 h2o_c = 1 adn_c + 1 hcys_L_c | 1 S_Adenosyl_L_homocysteine + 1 H2O = 1 Adenosine + 1 L_Homocysteine |
| **33** | METAT | 1 atp_c + 1 h2o_c + 1 met_L_c = 1 amet_c + 1 pi_c + 1 ppi_c | 1 ATP + 1 H2O + 1 L_Methionine = 1 S_Adenosyl_L_methionine + 1 Phosphate + 1 Diphosphate |
| **34** | ARGN | 1 arg_L_c + 1 h2o_c = 1 orn_c + 1 urea_c | 1 L_Arginine + 1 H2O = 1 Ornithine + 1 Urea |
| **35** | HISD | 1 his_L_c = 1 nh4_c + 1 urcan_c | 1 L_Histidine = 1 Ammonium + 1 Urocanate |
| **36** | URCN | 1 h2o_c + 1 urcan_c = 1 _4izp_c | 1 H2O + 1 Urocanate = 1 _4_Imidazolone_5_propanoate |
| **37** | ADSL1 | 1 dcamp_c = 1 amp_c + 1 fum_c | 1 N6__1_2_Dicarboxyethyl_AMP = 1 AMP + 1 Fumarate |
| **38** | ADSL2 | 1 _25aics_c = 1 aicar_c + 1 fum_c | 1 _S_2_5_Amino_1_(5_phospho_D_ribosyl)imidazole_4_carboxamido_succinate = 1 _5_Amino_1__5_Phospho_D_ribosylimidazole_4_carboxamide + 1 Fumarate |
| **39** | AICART | 1 _10fthf_c + 1 aicar_c = 1 fprica_c + 1 thf_c | 1 _10_Formyltetrahydrofolate + 1 _5_Amino_1__5_Phospho_D_ribosylimidazole_4_carboxamide = 1 _5_Formamido_1__5_phospho_D_ribosylimidazole_4_carboxamide + 1 _5_6_7_8_Tetrahydrofolate |
| **40** | IMPC | 1 h2o_c + 1 imp_c = 1 fprica_c | 1 H2O + 1 IMP = 1 _5_Formamido_1__5_phospho_D_ribosylimidazole_4_carboxamide |
| **41** | IMPD | 1 h2o_c + 1 imp_c + 1 nad_c = 1 h_c + 1 nadh_c + 1 xmp_c | 1 H2O + 1 IMP + 1 Nicotinamideadeninedinucleotide = 1 H+ + 1 Nicotinamideadeninedinucleotide_reduced + 1 Xanthosine5_phosphate |
| **42** | CTPS2 | 1 atp_c + 1 gln_L_c + 1 h2o_c + 1 utp_c = 1 adp_c + 1 ctp_c + 1 glu_L_c + 2 h_c + 1 pi_c | 1 ATP + 1 L_Glutamine + 1 H2O + 1 UTP = 1 ADP + 1 CTP + 1 L_Glutamate + 2 H+ + 1 Phosphate |
| **43** | DHORTS | 1 dhor_S_c + 1 h2o_c = 1 cbasp_c + 1 h_c | 1 _S_Dihydroorotate + 1 H2O = 1 N_Carbamoyl_L_aspartate + 1 H+ |
| **44** | OMPDC | 1 h_c + 1 orot5p_c = 1 co2_c + 1 ump_c | 1 H+ + 1 Orotidine5_phosphate = 1 CO2 + 1 UMP |
| **45** | ORPT | 1 orot5p_c + 1 ppi_c = 1 orot_c + 1 prpp_c | 1 Orotidine5_phosphate + 1 Diphosphate = 1 Orotate + 1 _5_Phospho_alpha_D_ribose1_diphosphate |
| **46** | ADNK1 | 1 adn_c + 1 atp_c = 1 adp_c + 1 amp_c + 1 h_c | 1 Adenosine + 1 ATP = 1 ADP + 1 AMP + 1 H+ |
| **47** | ADK1 | 1 amp_c + 1 atp_c = 2 adp_c | 1 AMP + 1 ATP = 2 ADP |
| **48** | CYTK1 | 1 atp_c + 1 cmp_c = 1 adp_c + 1 cdp_c | 1 ATP + 1 CMP = 1 ADP + 1 CDP |
| **49** | CYTK2 | 1 atp_c + 1 dcmp_c = 1 adp_c + 1 dcdp_c | 1 ATP + 1 dCMP = 1 ADP + 1 dCDP |
| **50** | UMPK | 1 atp_c + 1 ump_c = 1 adp_c + 1 udp_c | 1 ATP + 1 UMP = 1 ADP + 1 UDP |
| **51** | GK1 | 1 atp_c + 1 gmp_c = 1 adp_c + 1 gdp_c | 1 ATP + 1 GMP = 1 ADP + 1 GDP |
| **52** | DTMPK | 1 atp_c + 1 dtmp_c = 1 adp_c + 1 dtdp_c | 1 ATP + 1 dTMP = 1 ADP + 1 dTDP |
| **53** | NDPK1 | 1 atp_c + 1 gdp_c = 1 adp_c + 1 gtp_c | 1 ATP + 1 GDP = 1 ADP + 1 GTP |
| **54** | NDPK2 | 1 atp_c + 1 udp_c = 1 adp_c + 1 utp_c | 1 ATP + 1 UDP = 1 ADP + 1 UTP |
| **55** | NDPK3 | 1 atp_c + 1 cdp_c = 1 adp_c + 1 ctp_c | 1 ATP + 1 CDP = 1 ADP + 1 CTP |
| **56** | NDPK4 | 1 atp_c + 1 dtdp_c = 1 adp_c + 1 dttp_c | 1 ATP + 1 dTDP = 1 ADP + 1 dTTP |
| **57** | NDPK5 | 1 atp_c + 1 dgdp_c = 1 adp_c + 1 dgtp_c | 1 ATP + 1 dGDP = 1 ADP + 1 dGTP |
| **58** | NDPK7 | 1 atp_c + 1 dcdp_c = 1 adp_c + 1 dctp_c | 1 ATP + 1 dCDP = 1 ADP + 1 dCTP |
| **59** | NDPK8 | 1 atp_c + 1 dadp_c = 1 adp_c + 1 datp_c | 1 ATP + 1 dADP = 1 ADP + 1 dATP |
| **60** | RNDR1 | 1 adp_c + 1 trdrd_c = 1 dadp_c + 1 h2o_c + 1 trdox_c | 1 ADP + 1 Reducedthioredoxin = 1 dADP + 1 H2O + 1 Oxidizedthioredoxin |
| **61** | RNDR2 | 1 gdp_c + 1 trdrd_c = 1 dgdp_c + 1 h2o_c + 1 trdox_c | 1 GDP + 1 Reducedthioredoxin = 1 dGDP + 1 H2O + 1 Oxidizedthioredoxin |
| **62** | RNDR3 | 1 cdp_c + 1 trdrd_c = 1 dcdp_c + 1 h2o_c + 1 trdox_c | 1 CDP + 1 Reducedthioredoxin = 1 dCDP + 1 H2O + 1 Oxidizedthioredoxin |
| **63** | TMDS | 1 dump_c + 1 mlthf_c = 1 dhf_c + 1 dtmp_c | 1 dUMP + 1 _5_10_Methylenetetrahydrofolate = 1 _7_8_Dihydrofolate + 1 dTMP |
| **64** | EX_glc_e | 1 glc_D_e = 1 glc_D_b | 1 D_Glucose = 1 D_Glucose |
| **65** | EX_h2o_e | 1 h2o_e = 1 h2o_b | 1 H2O = 1 H2O |
| **66** | EX_pi_e | 1 pi_e = 1 pi_b | 1 Phosphate = 1 Phosphate |
| **67** | EX_h_e | 1 h_e = 1 h_b | 1 H+ = 1 H+ |
| **68** | DHFR | 1 dhf_c + 1 h_c + 1 nadph_c = 1 nadp_c + 1 thf_c | 1 _7_8_Dihydrofolate + 1 H+ + 1 Nicotinamideadeninedinucleotidephosphate_reduced = 1 Nicotinamideadeninedinucleotidephosphate + 1 _5_6_7_8_Tetrahydrofolate |
| **69** | ACCOAC | 1 accoa_c + 1 atp_c + 1 hco3_c = 1 adp_c + 1 h_c + 1 malcoa_c + 1 pi_c | 1 Acetyl_CoA + 1 ATP + 1 Bicarbonate = 1 ADP + 1 H+ + 1 Malonyl_CoA + 1 Phosphate |
| **70** | ACACT1 | 2 accoa_c = 1 aacoa_c + 1 coa_c | 2 Acetyl_CoA = 1 Acetoacetyl_CoA + 1 CoenzymeA |
| **71** | PDHm | 1 coa_m + 1 nad_m + 1 pyr_m = 1 accoa_m + 1 co2_m + 1 nadh_m | 1 CoenzymeA + 1 Nicotinamideadeninedinucleotide + 1 Pyruvate = 1 Acetyl_CoA + 1 CO2 + 1 Nicotinamideadeninedinucleotide_reduced |
| **72** | CSm | 1 accoa_m + 1 h2o_m + 1 oaa_m = 1 cit_m + 1 coa_m + 1 h_m | 1 Acetyl_CoA + 1 H2O + 1 Oxaloacetate = 1 Citrate + 1 CoenzymeA + 1 H+ |
| **73** | ACONTm | 1 cit_m = 1 icit_m | 1 Citrate = 1 Isocitrate |
| **74** | ICDHxm | 1 icit_m + 1 nad_m = 1 akg_m + 1 co2_m + 1 nadh_m | 1 Isocitrate + 1 Nicotinamideadeninedinucleotide = 1 _2_Oxoglutarate + 1 CO2 + 1 Nicotinamideadeninedinucleotide_reduced |
| **75** | MDH | 1 mal_L_c + 1 nad_c = 1 h_c + 1 nadh_c + 1 oaa_c | 1 L_Malate + 1 Nicotinamideadeninedinucleotide = 1 H+ + 1 Nicotinamideadeninedinucleotide_reduced + 1 Oxaloacetate |
| **76** | MDHm | 1 mal_L_m + 1 nad_m = 1 h_m + 1 nadh_m + 1 oaa_m | 1 L_Malate + 1 Nicotinamideadeninedinucleotide = 1 H+ + 1 Nicotinamideadeninedinucleotide_reduced + 1 Oxaloacetate |
| **77** | FUMm | 1 fum_m + 1 h2o_m = 1 mal_L_m | 1 Fumarate + 1 H2O = 1 L_Malate |
| **78** | EX_gln_L_e | 1 gln_L_e = 1 gln_L_b | 1 L_Glutamine = 1 L_Glutamine |
| **79** | EX_so4_e | 1 so4_e = 1 so4_b | 1 Sulfate = 1 Sulfate |
| **80** | EX_o2_e | 1 o2_e = 1 o2_b | 1 O2 = 1 O2 |
| **81** | EX_co2_e | 1 co2_e = 1 co2_b | 1 CO2 = 1 CO2 |
| **82** | EX_ala_L_e | 1 ala_L_e = 1 ala_L_b | 1 L_Alanine = 1 L_Alanine |
| **83** | EX_arg_L_e | 1 arg_L_e = 1 arg_L_b | 1 L_Arginine = 1 L_Arginine |
| **84** | EX_asn_L_e | 1 asn_L_e = 1 asn_L_b | 1 L_Asparagine = 1 L_Asparagine |
| **85** | EX_asp_L_e | 1 asp_L_e = 1 asp_L_b | 1 L_Aspartate = 1 L_Aspartate |
| **86** | EX_glu_L_e | 1 glu_L_e = 1 glu_L_b | 1 L_Glutamate = 1 L_Glutamate |
| **87** | EX_his_L_e | 1 his_L_e = 1 his_L_b | 1 L_Histidine = 1 L_Histidine |
| **88** | EX_ile_L_e | 1 ile_L_e = 1 ile_L_b | 1 L_Isoleucine = 1 L_Isoleucine |
| **89** | EX_lac_L_e | 1 lac_L_e = 1 lac_L_b | 1 L_Lactate = 1 L_Lactate |
| **90** | EX_leu_L_e | 1 leu_L_e = 1 leu_L_b | 1 L_Leucine = 1 L_Leucine |
| **91** | EX_phe_L_e | 1 phe_L_e = 1 phe_L_b | 1 L_Phenylalanine = 1 L_Phenylalanine |
| **92** | EX_pro_L_e | 1 pro_L_e = 1 pro_L_b | 1 L_Proline = 1 L_Proline |
| **93** | EX_thr_L_e | 1 thr_L_e = 1 thr_L_b | 1 L_Threonine = 1 L_Threonine |
| **94** | EX_tyr_L_e | 1 tyr_L_e = 1 tyr_L_b | 1 L_Tyrosine = 1 L_Tyrosine |
| **95** | EX_val_L_e | 1 val_L_e = 1 val_L_b | 1 L_Valine = 1 L_Valine |
| **96** | H2Ot | 1 h2o_e = 1 h2o_c | 1 H2O = 1 H2O |
| **97** | CO2tm | 1 co2_c = 1 co2_m | 1 CO2 = 1 CO2 |
| **98** | PIt2m | 1 h_c + 1 pi_c = 1 h_m + 1 pi_m | 1 H+ + 1 Phosphate = 1 H+ + 1 Phosphate |
| **99** | PYRt2m | 1 h_c + 1 pyr_c = 1 h_m + 1 pyr_m | 1 H+ + 1 Pyruvate = 1 H+ + 1 Pyruvate |
| **100** | H2Otm | 1 h2o_c = 1 h2o_m | 1 H2O = 1 H2O |
| **101** | EX_nh4_e | 1 nh4_e = 1 nh4_b | 1 Ammonium = 1 Ammonium |
| **102** | PSERT | 1 _3php_c + 1 glu_L_c = 1 akg_c + 1 pser_L_c | 1 _3_Phosphohydroxypyruvate + 1 L_Glutamate = 1 _2_Oxoglutarate + 1 O_Phospho_L_serine |
| **103** | SUCOASm | 1 atp_m + 1 coa_m + 1 succ_m = 1 adp_m + 1 pi_m + 1 succoa_m | 1 ATP + 1 CoenzymeA + 1 Succinate = 1 ADP + 1 Phosphate + 1 Succinyl_CoA |
| **104** | MALtm | 1 mal_L_c + 1 pi_m = 1 mal_L_m + 1 pi_c | 1 L_Malate + 1 Phosphate = 1 L_Malate + 1 Phosphate |
| **105** | HCO3Em | 1 co2_m + 1 h2o_m = 1 h_m + 1 hco3_m | 1 CO2 + 1 H2O = 1 H+ + 1 Bicarbonate |
| **106** | O2tm | 1 o2_c = 1 o2_m | 1 O2 = 1 O2 |
| **107** | CITtam | 1 cit_c + 1 mal_L_m = 1 cit_m + 1 mal_L_c | 1 Citrate + 1 L_Malate = 1 Citrate + 1 L_Malate |
| **108** | EX_met_L_e | 1 met_L_e = 1 met_L_b | 1 L_Methionine = 1 L_Methionine |
| **109** | H2CO3D | 1 co2_c + 1 h2o_c = 1 h2co3_c | 1 CO2 + 1 H2O = 1 carbonicacid |
| **110** | SUCD1m | 1 fad_m + 1 succ_m = 1 fadh2_m + 1 fum_m | 1 Flavinadeninedinucleotideoxidized + 1 Succinate = 1 Flavinadeninedinucleotidereduced + 1 Fumarate |
| **111** | G6PDH2r | 1 g6p_c + 1 nadp_c = 1 _6pgl_c + 1 h_c + 1 nadph_c | 1 D_Glucose6_phosphate + 1 Nicotinamideadeninedinucleotidephosphate = 1 _6_phospho_D_glucono_1_5_lactone + 1 H+ + 1 Nicotinamideadeninedinucleotidephosphate_reduced |
| **112** | ORNDC | 1 h_c + 1 orn_c = 1 co2_c + 1 ptrc_c | 1 H+ + 1 Ornithine = 1 CO2 + 1 Putrescine |
| **113** | ABUTD | 1 _4abutn_c + 1 h2o_c + 1 nad_c = 1 _4abut_c + 2 h_c + 1 nadh_c | 1 _4_Aminobutanal + 1 H2O + 1 Nicotinamideadeninedinucleotide = 1 _4_Aminobutanoate + 2 H+ + 1 Nicotinamideadeninedinucleotide_reduced |
| **114** | CO2t | 1 co2_e = 1 co2_c | 1 CO2 = 1 CO2 |
| **115** | O2t | 1 o2_e = 1 o2_c | 1 O2 = 1 O2 |
| **116** | EX_cys_L_e | 1 cys_L_e = 1 cys_L_b | 1 L_Cysteine = 1 L_Cysteine |
| **117** | EX_gly_e | 1 gly_e = 1 gly_b | 1 Glycine = 1 Glycine |
| **118** | EX_lys_L_e | 1 lys_L_e = 1 lys_L_b | 1 L_Lysine = 1 L_Lysine |
| **119** | EX_ser_L_e | 1 ser_L_e = 1 ser_L_b | 1 L_Serine = 1 L_Serine |
| **120** | EX_trp_L_e | 1 trp_L_e = 1 trp_L_b | 1 L_Tryptophan = 1 L_Tryptophan |
| **121** | EX_urea_e | 1 urea_e = 1 urea_b | 1 Urea = 1 Urea |
| **122** | GMPS2 | 1 atp_c + 1 gln_L_c + 1 h2o_c + 1 xmp_c = 1 amp_c + 1 glu_L_c + 1 gmp_c + 2 h_c + 1 ppi_c | 1 ATP + 1 L_Glutamine + 1 H2O + 1 Xanthosine5_phosphate = 1 AMP + 1 L_Glutamate + 1 GMP + 2 H+ + 1 Diphosphate |
| **123** | L_LACt2r | 1 h_e + 1 lac_L_e = 1 h_c + 1 lac_L_c | 1 H+ + 1 L_Lactate = 1 H+ + 1 L_Lactate |
| **124** | AIRCr | 1 air_c + 1 co2_c = 1 _5aizc_c + 1 h_c | 1 _5_amino_1__5_phospho_D_ribosylimidazole + 1 CO2 = 1 _5_amino_1__5_phospho_D_ribosylimidazole_4_carboxylate + 1 H+ |
| **125** | GHMT2r | 1 ser_L_c + 1 thf_c = 1 gly_c + 1 h2o_c + 1 mlthf_c | 1 L_Serine + 1 _5_6_7_8_Tetrahydrofolate = 1 Glycine + 1 H2O + 1 _5_10_Methylenetetrahydrofolate |
| **126** | CYSTS | 1 hcys_L_c + 1 ser_L_c = 1 cyst_L_c + 1 h2o_c | 1 L_Homocysteine + 1 L_Serine = 1 L_Cystathionine + 1 H2O |
| **127** | CYSTGL | 1 cyst_L_c + 1 h2o_c = 1 _2obut_c + 1 cys_L_c + 1 nh4_c | 1 L_Cystathionine + 1 H2O = 1 _2_Oxobutanoate + 1 L_Cysteine + 1 Ammonium |
| **128** | LEUTAm | 1 akg_m + 1 leu_L_m = 1 _4mop_m + 1 glu_L_m | 1 _2_Oxoglutarate + 1 L_Leucine = 1 _4_Methyl_2_oxopentanoate + 1 L_Glutamate |
| **129** | ILETAm | 1 akg_m + 1 ile_L_m = 1 _3mop_m + 1 glu_L_m | 1 _2_Oxoglutarate + 1 L_Isoleucine = 1 _S_3_Methyl_2_oxopentanoate + 1 L_Glutamate |
| **130** | TRPO2 | 1 o2_c + 1 trp_L_c = 1 Lfmkynr_c | 1 O2 + 1 L_Tryptophan = 1 L_Formylkynurenine |
| **131** | PCLAD | 1 cmusa_c + 1 h_c = 1 am6sa_c + 1 co2_c | 1 _2_Amino_3_carboxymuconatesemialdehyde + 1 H+ = 1 _2_Aminomuconate6_semialdehyde + 1 CO2 |
| **132** | AM6SAD | 1 am6sa_c + 1 h2o_c + 1 nad_c = 1 amuco_c + 2 h_c + 1 nadh_c | 1 _2_Aminomuconate6_semialdehyde + 1 H2O + 1 Nicotinamideadeninedinucleotide = 1 _2_Aminomuconate + 2 H+ + 1 Nicotinamideadeninedinucleotide_reduced |
| **133** | PROtm | 1 pro_L_c = 1 pro_L_m | 1 L_Proline = 1 L_Proline |
| **134** | GLUt2m | 1 glu_L_c + 1 h_c = 1 glu_L_m + 1 h_m | 1 L_Glutamate + 1 H+ = 1 L_Glutamate + 1 H+ |
| **135** | ALAt2r | 1 ala_L_e + 1 h_e = 1 ala_L_c + 1 h_c | 1 L_Alanine + 1 H+ = 1 L_Alanine + 1 H+ |
| **136** | GLYt2r | 1 gly_e + 1 h_e = 1 gly_c + 1 h_c | 1 Glycine + 1 H+ = 1 Glycine + 1 H+ |
| **137** | PROt2r | 1 h_e + 1 pro_L_e = 1 h_c + 1 pro_L_c | 1 H+ + 1 L_Proline = 1 H+ + 1 L_Proline |
| **138** | COAtp | 1 coa_c = 1 coa_x | 1 CoenzymeA = 1 CoenzymeA |
| **139** | _4ABUTtm | 1 _4abut_c = 1 _4abut_m | 1 _4_Aminobutanoate = 1 _4_Aminobutanoate |
| **140** | SERPT | 1 h_c + 1 pmtcoa_c + 1 ser_L_c = 1 _3dsphgn_c + 1 co2_c + 1 coa_c | 1 H+ + 1 Palmitoyl_CoA_n_C16_0CoA + 1 L_Serine = 1 _3_Dehydrosphinganine + 1 CO2 + 1 CoenzymeA |
| **141** | _3DSPHR | 1 _3dsphgn_c + 1 h_c + 1 nadph_c = 1 nadp_c + 1 sphgn_c | 1 _3_Dehydrosphinganine + 1 H+ + 1 Nicotinamideadeninedinucleotidephosphate_reduced = 1 Nicotinamideadeninedinucleotidephosphate + 1 Sphinganine |
| **142** | FAS100COA | 3 h_c + 1 malcoa_c + 2 nadph_c + 1 occoa_c = 1 co2_c + 1 coa_c + 1 dcacoa_c + 1 h2o_c + 2 nadp_c | 3 H+ + 1 Malonyl_CoA + 2 Nicotinamideadeninedinucleotidephosphate_reduced + 1 Octanoyl_CoA_n_C8_0CoA = 1 CO2 + 1 CoenzymeA + 1 Decanoyl_CoA_n_C10_0CoA + 1 H2O + 2 Nicotinamideadeninedinucleotidephosphate |
| **143** | FAS80COA_L | 1 accoa_c + 9 h_c + 3 malcoa_c + 6 nadph_c = 3 co2_c + 3 coa_c + 3 h2o_c + 6 nadp_c + 1 occoa_c | 1 Acetyl_CoA + 9 H+ + 3 Malonyl_CoA + 6 Nicotinamideadeninedinucleotidephosphate_reduced = 3 CO2 + 3 CoenzymeA + 3 H2O + 6 Nicotinamideadeninedinucleotidephosphate + 1 Octanoyl_CoA_n_C8_0CoA |
| **144** | FAS120COA | 1 dcacoa_c + 3 h_c + 1 malcoa_c + 2 nadph_c = 1 co2_c + 1 coa_c + 1 ddcacoa_c + 1 h2o_c + 2 nadp_c | 1 Decanoyl_CoA_n_C10_0CoA + 3 H+ + 1 Malonyl_CoA + 2 Nicotinamideadeninedinucleotidephosphate_reduced = 1 CO2 + 1 CoenzymeA + 1 Dodecanoyl_CoA_n_C12_0CoA + 1 H2O + 2 Nicotinamideadeninedinucleotidephosphate |
| **145** | FAS140COA | 1 ddcacoa_c + 3 h_c + 1 malcoa_c + 2 nadph_c = 1 co2_c + 1 coa_c + 1 h2o_c + 2 nadp_c + 1 tdcoa_c | 1 Dodecanoyl_CoA_n_C12_0CoA + 3 H+ + 1 Malonyl_CoA + 2 Nicotinamideadeninedinucleotidephosphate_reduced = 1 CO2 + 1 CoenzymeA + 1 H2O + 2 Nicotinamideadeninedinucleotidephosphate + 1 Tetradecanoyl_CoA_n_C14_0CoA |
| **146** | FAS160COA | 3 h_c + 1 malcoa_c + 2 nadph_c + 1 tdcoa_c = 1 co2_c + 1 coa_c + 1 h2o_c + 2 nadp_c + 1 pmtcoa_c | 3 H+ + 1 Malonyl_CoA + 2 Nicotinamideadeninedinucleotidephosphate_reduced + 1 Tetradecanoyl_CoA_n_C14_0CoA = 1 CO2 + 1 CoenzymeA + 1 H2O + 2 Nicotinamideadeninedinucleotidephosphate + 1 Palmitoyl_CoA_n_C16_0CoA |
| **147** | SQLEr | 1 h_r + 1 nadph_r + 1 o2_r + 1 sql_r = 1 Ssq23epx_r + 1 h2o_r + 1 nadp_r | 1 H+ + 1 Nicotinamideadeninedinucleotidephosphate_reduced + 1 O2 + 1 Squalene = 1 _S_Squalene_2_3_epoxide + 1 H2O + 1 Nicotinamideadeninedinucleotidephosphate |
| **148** | CO2tp | 1 co2_c = 1 co2_x | 1 CO2 = 1 CO2 |
| **149** | O2ter | 1 o2_c = 1 o2_r | 1 O2 = 1 O2 |
| **150** | H2Oter | 1 h2o_c = 1 h2o_r | 1 H2O = 1 H2O |
| **151** | CO2ter | 1 co2_c = 1 co2_r | 1 CO2 = 1 CO2 |
| **152** | MCITS | 1 h2o_c + 1 oaa_c + 1 ppcoa_c = 1 _2mcit_c + 1 coa_c + 1 h_c | 1 H2O + 1 Oxaloacetate + 1 Propanoyl_CoA = 1 _2_Methylcitrate + 1 CoenzymeA + 1 H+ |
| **153** | AMCOXO | 1 amuco_c + 1 h_c + 1 h2o_c + 1 nadph_c = 1 _2oxoadp_c + 1 nadp_c + 1 nh4_c | 1 _2_Aminomuconate + 1 H+ + 1 H2O + 1 Nicotinamideadeninedinucleotidephosphate_reduced = 1 _2_Oxoadipate + 1 Nicotinamideadeninedinucleotidephosphate + 1 Ammonium |
| **154** | HKYNH | 1 h2o_c + 1 hLkynr_c = 1 _3hanthrn_c + 1 ala_L_c | 1 H2O + 1 _3_Hydroxy_L_kynurenine = 1 _3_Hydroxyanthranilate + 1 L_Alanine |
| **155** | _3HAO | 1 _3hanthrn_c + 1 o2_c = 1 cmusa_c + 1 h_c | 1 _3_Hydroxyanthranilate + 1 O2 = 1 _2_Amino_3_carboxymuconatesemialdehyde + 1 H+ |
| **156** | MACACI | 1 _4mlacac_c = 1 _4fumacac_c | 1 _4_Maleylacetoacetate = 1 _4_Fumarylacetoacetate |
| **157** | FUMAC | 1 _4fumacac_c + 1 h2o_c = 1 acac_c + 1 fum_c + 1 h_c | 1 _4_Fumarylacetoacetate + 1 H2O = 1 Acetoacetate + 1 Fumarate + 1 H+ |
| **158** | HGNTOR | 1 hgentis_c + 1 o2_c = 1 _4mlacac_c + 1 h_c | 1 Homogentisate + 1 O2 = 1 _4_Maleylacetoacetate + 1 H+ |
| **159** | FKYNH | 1 Lfmkynr_c + 1 h2o_c = 1 Lkynr_c + 1 for_c + 1 h_c | 1 L_Formylkynurenine + 1 H2O = 1 L_Kynurenine + 1 Formate + 1 H+ |
| **160** | CHLPCTD | 1 cholp_c + 1 ctp_c + 1 h_c = 1 cdpchol_c + 1 ppi_c | 1 Cholinephosphate + 1 CTP + 1 H+ = 1 CDPcholine + 1 Diphosphate |
| **161** | CHOLK | 1 atp_c + 1 chol_c = 1 adp_c + 1 cholp_c + 1 h_c | 1 ATP + 1 Choline = 1 ADP + 1 Cholinephosphate + 1 H+ |
| **162** | _34HPPOR | 1 _34hpp_c + 1 o2_c = 1 co2_c + 1 hgentis_c | 1 _3__4_Hydroxyphenylpyruvate + 1 O2 = 1 CO2 + 1 Homogentisate |
| **163** | SPODM | 2 h_c + 2 o2s_c = 1 h2o2_c + 1 o2_c | 2 H+ + 2 Superoxideanion = 1 Hydrogenperoxide + 1 O2 |
| **164** | SERt4 | 1 na1_e + 1 ser_L_e = 1 na1_c + 1 ser_L_c | 1 Sodium + 1 L_Serine = 1 Sodium + 1 L_Serine |
| **165** | THRt4 | 1 na1_e + 1 thr_L_e = 1 na1_c + 1 thr_L_c | 1 Sodium + 1 L_Threonine = 1 Sodium + 1 L_Threonine |
| **166** | ATPtm | 1 adp_c + 1 atp_m = 1 adp_m + 1 atp_c | 1 ADP + 1 ATP = 1 ADP + 1 ATP |
| **167** | ORNt3m | 1 h_c + 1 orn_m = 1 h_m + 1 orn_c | 1 H+ + 1 Ornithine = 1 H+ + 1 Ornithine |
| **168** | ADSS | 1 asp_L_c + 1 gtp_c + 1 imp_c = 1 dcamp_c + 1 gdp_c + 2 h_c + 1 pi_c | 1 L_Aspartate + 1 GTP + 1 IMP = 1 N6__1_2_Dicarboxyethyl_AMP + 1 GDP + 2 H+ + 1 Phosphate |
| **169** | GLNS | 1 atp_c + 1 glu_L_c + 1 nh4_c = 1 adp_c + 1 gln_L_c + 1 h_c + 1 pi_c | 1 ATP + 1 L_Glutamate + 1 Ammonium = 1 ADP + 1 L_Glutamine + 1 H+ + 1 Phosphate |
| **170** | GLUPRT | 1 gln_L_c + 1 h2o_c + 1 prpp_c = 1 glu_L_c + 1 ppi_c + 1 pram_c | 1 L_Glutamine + 1 H2O + 1 _5_Phospho_alpha_D_ribose1_diphosphate = 1 L_Glutamate + 1 Diphosphate + 1 _5_Phospho_beta_D_ribosylamine |
| **171** | PRAGSr | 1 atp_c + 1 gly_c + 1 pram_c = 1 adp_c + 1 gar_c + 1 h_c + 1 pi_c | 1 ATP + 1 Glycine + 1 _5_Phospho_beta_D_ribosylamine = 1 ADP + 1 N1__5_Phospho_D_ribosylglycinamide + 1 H+ + 1 Phosphate |
| **172** | PRFGS | 1 atp_c + 1 fgam_c + 1 gln_L_c + 1 h2o_c = 1 adp_c + 1 fpram_c + 1 glu_L_c + 1 h_c + 1 pi_c | 1 ATP + 1 N2_Formyl_N1__5_phospho_D_ribosylglycinamide + 1 L_Glutamine + 1 H2O = 1 ADP + 1 _2__Formamido_N1_(5_phospho_D_ribosyl)acetamidine + 1 L_Glutamate + 1 H+ + 1 Phosphate |
| **173** | KYN3OX | 1 Lkynr_c + 1 h_c + 1 nadph_c + 1 o2_c = 1 h2o_c + 1 hLkynr_c + 1 nadp_c | 1 L_Kynurenine + 1 H+ + 1 Nicotinamideadeninedinucleotidephosphate_reduced + 1 O2 = 1 H2O + 1 _3_Hydroxy_L_kynurenine + 1 Nicotinamideadeninedinucleotidephosphate |
| **174** | GARFT | 1 _10fthf_c + 1 gar_c = 1 fgam_c + 1 h_c + 1 thf_c | 1 _10_Formyltetrahydrofolate + 1 N1__5_Phospho_D_ribosylglycinamide = 1 N2_Formyl_N1__5_phospho_D_ribosylglycinamide + 1 H+ + 1 _5_6_7_8_Tetrahydrofolate |
| **175** | PRAIS | 1 atp_c + 1 fpram_c = 1 adp_c + 1 air_c + 2 h_c + 1 pi_c | 1 ATP + 1 _2__Formamido_N1_(5_phospho_D_ribosyl)acetamidine = 1 ADP + 1 _5_amino_1__5_phospho_D_ribosylimidazole + 2 H+ + 1 Phosphate |
| **176** | PRASCS | 1 _5aizc_c + 1 asp_L_c + 1 atp_c = 1 _25aics_c + 1 adp_c + 1 h_c + 1 pi_c | 1 _5_amino_1__5_phospho_D_ribosylimidazole_4_carboxylate + 1 L_Aspartate + 1 ATP = 1 _S_2_5_Amino_1_(5_phospho_D_ribosyl)imidazole_4_carboxamido_succinate + 1 ADP + 1 H+ + 1 Phosphate |
| **177** | PROD2 | 1 fad_c + 1 pro_L_c = 1 _1pyr5c_c + 1 fadh2_c + 1 h_c | 1 Flavinadeninedinucleotideoxidized + 1 L_Proline = 1 _1_Pyrroline_5_carboxylate + 1 Flavinadeninedinucleotidereduced + 1 H+ |
| **178** | DCMPDA | 1 dcmp_c + 1 h_c + 1 h2o_c = 1 dump_c + 1 nh4_c | 1 dCMP + 1 H+ + 1 H2O = 1 dUMP + 1 Ammonium |
| **179** | G5SADrm | 1 glu5sa_m = 1 _1pyr5c_m + 1 h_m + 1 h2o_m | 1 L_Glutamate5_semialdehyde = 1 _1_Pyrroline_5_carboxylate + 1 H+ + 1 H2O |
| **180** | VALTAm | 1 akg_m + 1 val_L_m = 1 _3mob_m + 1 glu_L_m | 1 _2_Oxoglutarate + 1 L_Valine = 1 _3_Methyl_2_oxobutanoate + 1 L_Glutamate |
| **181** | AKGDm | 1 akg_m + 1 coa_m + 1 nad_m = 1 co2_m + 1 nadh_m + 1 succoa_m | 1 _2_Oxoglutarate + 1 CoenzymeA + 1 Nicotinamideadeninedinucleotide = 1 CO2 + 1 Nicotinamideadeninedinucleotide_reduced + 1 Succinyl_CoA |
| **182** | NADH2_u10m | 5 h_m + 1 nadh_m + 1 q10_m = 4 h_c + 1 nad_m + 1 q10h2_m | 5 H+ + 1 Nicotinamideadeninedinucleotide_reduced + 1 Ubiquinone_10 = 4 H+ + 1 Nicotinamideadeninedinucleotide + 1 Ubiquinol_10 |
| **183** | AKGMALtm | 1 akg_m + 1 mal_L_c = 1 akg_c + 1 mal_L_m | 1 _2_Oxoglutarate + 1 L_Malate = 1 _2_Oxoglutarate + 1 L_Malate |
| **184** | CATm | 2 h2o2_m = 2 h2o_m + 1 o2_m | 2 Hydrogenperoxide = 2 H2O + 1 O2 |
| **185** | HMGLm | 1 hmgcoa_m = 1 acac_m + 1 accoa_m | 1 Hydroxymethylglutaryl_CoA = 1 Acetoacetate + 1 Acetyl_CoA |
| **186** | PPCOACm | 1 atp_m + 1 hco3_m + 1 ppcoa_m = 1 adp_m + 1 h_m + 1 mmcoa_S_m + 1 pi_m | 1 ATP + 1 Bicarbonate + 1 Propanoyl_CoA = 1 ADP + 1 H+ + 1 _S_Methylmalonyl_CoA + 1 Phosphate |
| **187** | CYOR_u10m | 2 ficytC_m + 2 h_m + 1 q10h2_m = 2 focytC_m + 4 h_c + 1 q10_m | 2 Ferricytochromec + 2 H+ + 1 Ubiquinol_10 = 2 FerrocytochromeC + 4 H+ + 1 Ubiquinone_10 |
| **188** | ATPS4m | 1 adp_m + 4 h_c + 1 pi_m = 1 atp_m + 3 h_m + 1 h2o_m | 1 ADP + 4 H+ + 1 Phosphate = 1 ATP + 3 H+ + 1 H2O |
| **189** | CYOOm3 | 4 focytC_m + 7.92 h_m + 1 o2_m = 4 ficytC_m + 4 h_c + 1.96 h2o_m + 0.02 o2s_m | 4 FerrocytochromeC + 7.92 H+ + 1 O2 = 4 Ferricytochromec + 4 H+ + 1.96 H2O + 0.02 Superoxideanion |
| **190** | OCOAT1m | 1 acac_m + 1 succoa_m = 1 aacoa_m + 1 succ_m | 1 Acetoacetate + 1 Succinyl_CoA = 1 Acetoacetyl_CoA + 1 Succinate |
| **191** | PCm | 1 atp_m + 1 hco3_m + 1 pyr_m = 1 adp_m + 1 h_m + 1 oaa_m + 1 pi_m | 1 ATP + 1 Bicarbonate + 1 Pyruvate = 1 ADP + 1 H+ + 1 Oxaloacetate + 1 Phosphate |
| **192** | LYStm | 1 h_m + 1 lys_L_c = 1 h_c + 1 lys_L_m | 1 H+ + 1 L_Lysine = 1 H+ + 1 L_Lysine |
| **193** | THD1m | 1 h_c + 1 nadh_m + 1 nadp_m = 1 h_m + 1 nad_m + 1 nadph_m | 1 H+ + 1 Nicotinamideadeninedinucleotide_reduced + 1 Nicotinamideadeninedinucleotidephosphate = 1 H+ + 1 Nicotinamideadeninedinucleotide + 1 Nicotinamideadeninedinucleotidephosphate_reduced |
| **194** | ACACt2m | 1 acac_c + 1 h_c = 1 acac_m + 1 h_m | 1 Acetoacetate + 1 H+ = 1 Acetoacetate + 1 H+ |
| **195** | CYSO | 1 cys_L_c + 1 o2_c = 1 _3sala_c + 2 h_c | 1 L_Cysteine + 1 O2 = 1 _3_Sulfino_L_alanine + 2 H+ |
| **196** | H2CO3D2 | 1 h_c + 1 hco3_c = 1 h2co3_c | 1 H+ + 1 Bicarbonate = 1 carbonicacid |
| **197** | PIt7 | 3 na1_e + 1 pi_e = 3 na1_c + 1 pi_c | 3 Sodium + 1 Phosphate = 3 Sodium + 1 Phosphate |
| **198** | HMGCOASi | 1 aacoa_c + 1 accoa_c + 1 h2o_c = 1 coa_c + 1 h_c + 1 hmgcoa_c | 1 Acetoacetyl_CoA + 1 Acetyl_CoA + 1 H2O = 1 CoenzymeA + 1 H+ + 1 Hydroxymethylglutaryl_CoA |
| **199** | P5CRx | 1 _1pyr5c_c + 2 h_c + 1 nadh_c = 1 nad_c + 1 pro_L_c | 1 _1_Pyrroline_5_carboxylate + 2 H+ + 1 Nicotinamideadeninedinucleotide_reduced = 1 Nicotinamideadeninedinucleotide + 1 L_Proline |
| **200** | METS | 1 _5mthf_c + 1 hcys_L_c = 1 h_c + 1 met_L_c + 1 thf_c | 1 _5_Methyltetrahydrofolate + 1 L_Homocysteine = 1 H+ + 1 L_Methionine + 1 _5_6_7_8_Tetrahydrofolate |
| **201** | MTHFC | 1 h2o_c + 1 methf_c = 1 _10fthf_c + 1 h_c | 1 H2O + 1 _5_10_Methenyltetrahydrofolate = 1 _10_Formyltetrahydrofolate + 1 H+ |
| **202** | MTHFR3 | 2 h_c + 1 mlthf_c + 1 nadph_c = 1 _5mthf_c + 1 nadp_c | 2 H+ + 1 _5_10_Methylenetetrahydrofolate + 1 Nicotinamideadeninedinucleotidephosphate_reduced = 1 _5_Methyltetrahydrofolate + 1 Nicotinamideadeninedinucleotidephosphate |
| **203** | IZPN | 1 _4izp_c + 1 h2o_c = 1 forglu_c + 1 h_c | 1 _4_Imidazolone_5_propanoate + 1 H2O = 1 N_Formimidoyl_L_glutamate + 1 H+ |
| **204** | FUMtm | 1 fum_c + 1 pi_m = 1 fum_m + 1 pi_c | 1 Fumarate + 1 Phosphate = 1 Fumarate + 1 Phosphate |
| **205** | MMEm | 1 mmcoa_R_m = 1 mmcoa_S_m | 1 _R_Methylmalonyl_CoA = 1 _S_Methylmalonyl_CoA |
| **206** | GluForTx | 1 forglu_c + 1 h_c + 1 thf_c = 1 _5forthf_c + 1 glu_L_c | 1 N_Formimidoyl_L_glutamate + 1 H+ + 1 _5_6_7_8_Tetrahydrofolate = 1 _5_Formiminotetrahydrofolate + 1 L_Glutamate |
| **207** | ABTArm | 1 _4abut_m + 1 akg_m = 1 glu_L_m + 1 sucsal_m | 1 _4_Aminobutanoate + 1 _2_Oxoglutarate = 1 L_Glutamate + 1 Succinicsemialdehyde |
| **208** | ASPCTr | 1 asp_L_c + 1 cbp_c = 1 cbasp_c + 1 h_c + 1 pi_c | 1 L_Aspartate + 1 Carbamoylphosphate = 1 N_Carbamoyl_L_aspartate + 1 H+ + 1 Phosphate |
| **209** | _2OXOADOXm | 1 _2oxoadp_m + 1 coa_m + 1 nad_m = 1 co2_m + 1 glutcoa_m + 1 nadh_m | 1 _2_Oxoadipate + 1 CoenzymeA + 1 Nicotinamideadeninedinucleotide = 1 CO2 + 1 Glutaryl_CoA + 1 Nicotinamideadeninedinucleotide_reduced |
| **210** | GLUTCOADHm | 1 fad_m + 1 glutcoa_m + 1 h_m = 1 b2coa_m + 1 co2_m + 1 fadh2_m | 1 Flavinadeninedinucleotideoxidized + 1 Glutaryl_CoA + 1 H+ = 1 Crotonoyl_CoA + 1 CO2 + 1 Flavinadeninedinucleotidereduced |
| **211** | ECOAH1m | 1 _3hbcoa_m = 1 b2coa_m + 1 h2o_m | 1 _S_3_Hydroxybutanoyl_CoA = 1 Crotonoyl_CoA + 1 H2O |
| **212** | HACD1m | 1 aacoa_m + 1 h_m + 1 nadh_m = 1 _3hbcoa_m + 1 nad_m | 1 Acetoacetyl_CoA + 1 H+ + 1 Nicotinamideadeninedinucleotide_reduced = 1 _S_3_Hydroxybutanoyl_CoA + 1 Nicotinamideadeninedinucleotide |
| **213** | ACITL | 1 atp_c + 1 cit_c + 1 coa_c = 1 accoa_c + 1 adp_c + 1 oaa_c + 1 pi_c | 1 ATP + 1 Citrate + 1 CoenzymeA = 1 Acetyl_CoA + 1 ADP + 1 Oxaloacetate + 1 Phosphate |
| **214** | G5SDym | 1 glu5p_m + 1 h_m + 1 nadph_m = 1 glu5sa_m + 1 nadp_m + 1 pi_m | 1 L_Glutamate5_phosphate + 1 H+ + 1 Nicotinamideadeninedinucleotidephosphate_reduced = 1 L_Glutamate5_semialdehyde + 1 Nicotinamideadeninedinucleotidephosphate + 1 Phosphate |
| **215** | GLU5Km | 1 atp_m + 1 glu_L_m = 1 adp_m + 1 glu5p_m | 1 ATP + 1 L_Glutamate = 1 ADP + 1 L_Glutamate5_phosphate |
| **216** | ORNTArm | 1 akg_m + 1 orn_m = 1 glu_L_m + 1 glu5sa_m | 1 _2_Oxoglutarate + 1 Ornithine = 1 L_Glutamate + 1 L_Glutamate5_semialdehyde |
| **217** | HMGCOARx | 2 h_x + 1 hmgcoa_x + 2 nadph_x = 1 coa_x + 1 mev_R_x + 2 nadp_x | 2 H+ + 1 Hydroxymethylglutaryl_CoA + 2 Nicotinamideadeninedinucleotidephosphate_reduced = 1 CoenzymeA + 1 _R_Mevalonate + 2 Nicotinamideadeninedinucleotidephosphate |
| **218** | MEVK1x | 1 atp_x + 1 mev_R_x = 1 _5pmev_x + 1 adp_x + 1 h_x | 1 ATP + 1 _R_Mevalonate = 1 _R_5_Phosphomevalonate + 1 ADP + 1 H+ |
| **219** | PMEVKx | 1 _5pmev_x + 1 atp_x = 1 _5dpmev_x + 1 adp_x | 1 _R_5_Phosphomevalonate + 1 ATP = 1 _R_5_Diphosphomevalonate + 1 ADP |
| **220** | DPMVDx | 1 _5dpmev_x + 1 atp_x = 1 adp_x + 1 co2_x + 1 ipdp_x + 1 pi_x | 1 _R_5_Diphosphomevalonate + 1 ATP = 1 ADP + 1 CO2 + 1 Isopentenyldiphosphate + 1 Phosphate |
| **221** | IPDDIx | 1 ipdp_x = 1 dmpp_x | 1 Isopentenyldiphosphate = 1 Dimethylallyldiphosphate |
| **222** | GRTTx | 1 grdp_x + 1 ipdp_x = 1 frdp_x + 1 ppi_x | 1 Geranyldiphosphate + 1 Isopentenyldiphosphate = 1 Farnesyldiphosphate + 1 Diphosphate |
| **223** | DMATTx | 1 dmpp_x + 1 ipdp_x = 1 grdp_x + 1 ppi_x | 1 Dimethylallyldiphosphate + 1 Isopentenyldiphosphate = 1 Geranyldiphosphate + 1 Diphosphate |
| **224** | SQLSr | 2 frdp_r + 1 h_r + 1 nadph_r = 1 nadp_r + 2 ppi_r + 1 sql_r | 2 Farnesyldiphosphate + 1 H+ + 1 Nicotinamideadeninedinucleotidephosphate_reduced = 1 Nicotinamideadeninedinucleotidephosphate + 2 Diphosphate + 1 Squalene |
| **225** | LNSTLSr | 1 Ssq23epx_r = 1 lanost_r | 1 _S_Squalene_2_3_epoxide = 1 Lanosterol |
| **226** | LNS14DMr | 2 h_r + 1 lanost_r + 3 nadph_r + 3 o2_r = 1 _44mctr_r + 1 for_r + 4 h2o_r + 3 nadp_r | 2 H+ + 1 Lanosterol + 3 Nicotinamideadeninedinucleotidephosphate_reduced + 3 O2 = 1 _4_4_dimethylcholesta_8_14_24_trienol + 1 Formate + 4 H2O + 3 Nicotinamideadeninedinucleotidephosphate |
| **227** | C14STRr | 1 _44mctr_r + 1 h_r + 1 nadph_r = 1 _44mzym_r + 1 nadp_r | 1 _4_4_dimethylcholesta_8_14_24_trienol + 1 H+ + 1 Nicotinamideadeninedinucleotidephosphate_reduced = 1 _4_4_dimethylzymosterol + 1 Nicotinamideadeninedinucleotidephosphate |
| **228** | C4STMO1r | 1 _44mzym_r + 3 h_r + 3 nadph_r + 3 o2_r = 1 _4mzym_int1_r + 4 h2o_r + 3 nadp_r | 1 _4_4_dimethylzymosterol + 3 H+ + 3 Nicotinamideadeninedinucleotidephosphate_reduced + 3 O2 = 1 _4_Methylzymosterolintermediate1 + 4 H2O + 3 Nicotinamideadeninedinucleotidephosphate |
| **229** | C3STKR2r | 1 h_r + 1 nadph_r + 1 zym_int2_r = 1 nadp_r + 1 zymst_r | 1 H+ + 1 Nicotinamideadeninedinucleotidephosphate_reduced + 1 zymosterolintermediate2 = 1 Nicotinamideadeninedinucleotidephosphate + 1 zymosterol |
| **230** | EBP1r | 1 zymst_r = 1 chlstol_r | 1 zymosterol = 1 Cholesta_7_24_dien_3beta_ol |
| **231** | LSTO2r | 1 h_r + 1 lthstrl_r + 1 nadph_r + 1 o2_r = 1 _7dhchsterol_r + 2 h2o_r + 1 nadp_r | 1 H+ + 1 _5alpha_Cholest_7_en_3beta_ol + 1 Nicotinamideadeninedinucleotidephosphate_reduced + 1 O2 = 1 _7_Dehydrocholesterol + 2 H2O + 1 Nicotinamideadeninedinucleotidephosphate |
| **232** | DHCR72r | 1 _7dhchsterol_r + 1 h_r + 1 nadph_r = 1 chsterol_r + 1 nadp_r | 1 _7_Dehydrocholesterol + 1 H+ + 1 Nicotinamideadeninedinucleotidephosphate_reduced = 1 Cholesterol + 1 Nicotinamideadeninedinucleotidephosphate |
| **233** | DHCR242r | 1 chlstol_r + 1 fadh2_r = 1 fad_r + 1 lthstrl_r | 1 Cholesta_7_24_dien_3beta_ol + 1 Flavinadeninedinucleotidereduced = 1 Flavinadeninedinucleotideoxidized + 1 _5alpha_Cholest_7_en_3beta_ol |
| **234** | PIter | 1 pi_r = 1 pi_c | 1 Phosphate = 1 Phosphate |
| **235** | PPAer | 1 h2o_r + 1 ppi_r = 1 h_r + 2 pi_r | 1 H2O + 1 Diphosphate = 1 H+ + 2 Phosphate |
| **236** | ACOAD8m | 1 fad_m + 1 ivcoa_m = 1 _3mb2coa_m + 1 fadh2_m | 1 Flavinadeninedinucleotideoxidized + 1 Isovaleryl_CoA = 1 _3_Methylbut_2_enoyl_CoA + 1 Flavinadeninedinucleotidereduced |
| **237** | HIBDm | 1 _3hmp_m + 1 nad_m = 1 _2mop_m + 1 h_m + 1 nadh_m | 1 _3_Hydroxy_2_methylpropanoate + 1 Nicotinamideadeninedinucleotide = 1 _2_Methyl_3_oxopropanoate + 1 H+ + 1 Nicotinamideadeninedinucleotide_reduced |
| **238** | MCCCrm | 1 _3mb2coa_m + 1 atp_m + 1 hco3_m = 1 _3mgcoa_m + 1 adp_m + 1 h_m + 1 pi_m | 1 _3_Methylbut_2_enoyl_CoA + 1 ATP + 1 Bicarbonate = 1 _3_Methylglutaconyl_CoA + 1 ADP + 1 H+ + 1 Phosphate |
| **239** | MTHFD2 | 1 mlthf_c + 1 nad_c = 1 methf_c + 1 nadh_c | 1 _5_10_Methylenetetrahydrofolate + 1 Nicotinamideadeninedinucleotide = 1 _5_10_Methenyltetrahydrofolate + 1 Nicotinamideadeninedinucleotide_reduced |
| **240** | CEPTC | 1 cdpchol_c + 1 dag_hs_c = 1 cmp_c + 1 h_c + 1 pchol_hs_c | 1 CDPcholine + 1 diacylglycerol_homosapiens = 1 CMP + 1 H+ + 1 Phosphatidylcholine_homosapiens |
| **241** | PSSA1_hs | 1 pchol_hs_c + 1 ser_L_c = 1 chol_c + 1 ps_hs_c | 1 Phosphatidylcholine_homosapiens + 1 L_Serine = 1 Choline + 1 phosphatidylserine_homosapiens |
| **242** | CDS | 1 ctp_c + 1 h_c + 1 pa_hs_c = 1 cdpdag_hs_c + 1 ppi_c | 1 CTP + 1 H+ + 1 phosphatidicacid_homosapiens = 1 CDPdiacylglycerol_homosapiens + 1 Diphosphate |
| **243** | PTRCOX1 | 1 h2o_c + 1 o2_c + 1 ptrc_c = 1 _4abutn_c + 1 h2o2_c + 1 nh4_c | 1 H2O + 1 O2 + 1 Putrescine = 1 _4_Aminobutanal + 1 Hydrogenperoxide + 1 Ammonium |
| **244** | AACOAT | 1 acac_c + 1 atp_c + 1 coa_c = 1 aacoa_c + 1 amp_c + 1 ppi_c | 1 Acetoacetate + 1 ATP + 1 CoenzymeA = 1 Acetoacetyl_CoA + 1 AMP + 1 Diphosphate |
| **245** | PGPPT | 1 cdpdag_hs_c + 1 glyc3p_c = 1 cmp_c + 1 h_c + 1 pgp_hs_c | 1 CDPdiacylglycerol_homosapiens + 1 Glycerol3_phosphate = 1 CMP + 1 H+ + 1 phosphatidylglycerolphosphate_homosapiens |
| **246** | PGPP_hs | 1 h2o_c + 1 pgp_hs_c = 1 pglyc_hs_c + 1 pi_c | 1 H2O + 1 phosphatidylglycerolphosphate_homosapiens = 1 phosphatidylglycerol_homosapiens + 1 Phosphate |
| **247** | CLS_hs | 1 cdpdag_hs_c + 1 pglyc_hs_c = 1 clpn_hs_c + 1 cmp_c + 1 h_c | 1 CDPdiacylglycerol_homosapiens + 1 phosphatidylglycerol_homosapiens = 1 cardiolipin_homosapiens + 1 CMP + 1 H+ |
| **248** | SSALxm | 1 h2o_m + 1 nad_m + 1 sucsal_m = 2 h_m + 1 nadh_m + 1 succ_m | 1 H2O + 1 Nicotinamideadeninedinucleotide + 1 Succinicsemialdehyde = 2 H+ + 1 Nicotinamideadeninedinucleotide_reduced + 1 Succinate |
| **249** | SACCD3m | 1 akg_m + 1 h_m + 1 lys_L_m + 1 nadph_m = 1 h2o_m + 1 nadp_m + 1 saccrp_L_m | 1 _2_Oxoglutarate + 1 H+ + 1 L_Lysine + 1 Nicotinamideadeninedinucleotidephosphate_reduced = 1 H2O + 1 Nicotinamideadeninedinucleotidephosphate + 1 L_Saccharopine |
| **250** | SACCD4m | 1 h2o_m + 1 nadp_m + 1 saccrp_L_m = 1 L2aadp6sa_m + 1 glu_L_m + 1 h_m + 1 nadph_m | 1 H2O + 1 Nicotinamideadeninedinucleotidephosphate + 1 L_Saccharopine = 1 L_2_Aminoadipate6_semialdehyde + 1 L_Glutamate + 1 H+ + 1 Nicotinamideadeninedinucleotidephosphate_reduced |
| **251** | AATAi | 1 L2aadp_c + 1 akg_c = 1 _2oxoadp_c + 1 glu_L_c | 1 L_2_Aminoadipate + 1 _2_Oxoglutarate = 1 _2_Oxoadipate + 1 L_Glutamate |
| **252** | DHCRD1 | 1 dhcrm_hs_c + 1 nadp_c = 1 crm_hs_c + 1 h_c + 1 nadph_c | 1 dihydroceramide_homosapiens + 1 Nicotinamideadeninedinucleotidephosphate = 1 ceramide_homosapiens + 1 H+ + 1 Nicotinamideadeninedinucleotidephosphate_reduced |
| **253** | DAGK_hs | 1 atp_c + 1 dag_hs_c = 1 adp_c + 1 h_c + 1 pa_hs_c | 1 ATP + 1 diacylglycerol_homosapiens = 1 ADP + 1 H+ + 1 phosphatidicacid_homosapiens |
| **254** | OIVD1m | 1 _4mop_m + 1 coa_m + 1 nad_m = 1 co2_m + 1 ivcoa_m + 1 nadh_m | 1 _4_Methyl_2_oxopentanoate + 1 CoenzymeA + 1 Nicotinamideadeninedinucleotide = 1 CO2 + 1 Isovaleryl_CoA + 1 Nicotinamideadeninedinucleotide_reduced |
| **255** | OIVD2m | 1 _3mob_m + 1 coa_m + 1 nad_m = 1 co2_m + 1 ibcoa_m + 1 nadh_m | 1 _3_Methyl_2_oxobutanoate + 1 CoenzymeA + 1 Nicotinamideadeninedinucleotide = 1 CO2 + 1 Isobutyryl_CoA + 1 Nicotinamideadeninedinucleotide_reduced |
| **256** | OIVD3m | 1 _3mop_m + 1 coa_m + 1 nad_m = 1 _2mbcoa_m + 1 co2_m + 1 nadh_m | 1 _S_3_Methyl_2_oxopentanoate + 1 CoenzymeA + 1 Nicotinamideadeninedinucleotide = 1 _2_Methylbutanoyl_CoA + 1 CO2 + 1 Nicotinamideadeninedinucleotide_reduced |
| **257** | MGCHrm | 1 _3mgcoa_m + 1 h2o_m = 1 hmgcoa_m | 1 _3_Methylglutaconyl_CoA + 1 H2O = 1 Hydroxymethylglutaryl_CoA |
| **258** | ECOAH12m | 1 _2mp2coa_m + 1 h2o_m = 1 _3hibutcoa_m | 1 _2_Methylprop_2_enoyl_CoA + 1 H2O = 1 _S_3_Hydroxyisobutyryl_CoA |
| **259** | _3HBCOAHLm | 1 _3hibutcoa_m + 1 h2o_m = 1 _3hmp_m + 1 coa_m + 1 h_m | 1 _S_3_Hydroxyisobutyryl_CoA + 1 H2O = 1 _3_Hydroxy_2_methylpropanoate + 1 CoenzymeA + 1 H+ |
| **260** | ACOAD10m | 1 _2mbcoa_m + 1 fad_m = 1 _2mb2coa_m + 1 fadh2_m | 1 _2_Methylbutanoyl_CoA + 1 Flavinadeninedinucleotideoxidized = 1 trans_2_Methylbut_2_enoyl_CoA + 1 Flavinadeninedinucleotidereduced |
| **261** | ACOAD9m | 1 fad_m + 1 ibcoa_m = 1 _2mp2coa_m + 1 fadh2_m | 1 Flavinadeninedinucleotideoxidized + 1 Isobutyryl_CoA = 1 _2_Methylprop_2_enoyl_CoA + 1 Flavinadeninedinucleotidereduced |
| **262** | ECOAH9m | 1 _2mb2coa_m + 1 h2o_m = 1 _3hmbcoa_m | 1 trans_2_Methylbut_2_enoyl_CoA + 1 H2O = 1 _S_3_Hydroxy_2_methylbutyryl_CoA |
| **263** | HACD9m | 1 _3hmbcoa_m + 1 nad_m = 1 _2maacoa_m + 1 h_m + 1 nadh_m | 1 _S_3_Hydroxy_2_methylbutyryl_CoA + 1 Nicotinamideadeninedinucleotide = 1 _2_Methyl_3_acetoacetyl_CoA + 1 H+ + 1 Nicotinamideadeninedinucleotide_reduced |
| **264** | ACACT10m | 1 _2maacoa_m + 1 coa_m = 1 accoa_m + 1 ppcoa_m | 1 _2_Methyl_3_acetoacetyl_CoA + 1 CoenzymeA = 1 Acetyl_CoA + 1 Propanoyl_CoA |
| **265** | OBDHc | 1 _2obut_c + 1 coa_c + 1 nad_c = 1 co2_c + 1 nadh_c + 1 ppcoa_c | 1 _2_Oxobutanoate + 1 CoenzymeA + 1 Nicotinamideadeninedinucleotide = 1 CO2 + 1 Nicotinamideadeninedinucleotide_reduced + 1 Propanoyl_CoA |
| **266** | P5CRm | 1 _1pyr5c_m + 2 h_m + 1 nadph_m = 1 nadp_m + 1 pro_L_m | 1 _1_Pyrroline_5_carboxylate + 2 H+ + 1 Nicotinamideadeninedinucleotidephosphate_reduced = 1 Nicotinamideadeninedinucleotidephosphate + 1 L_Proline |
| **267** | _2OXOADPTm | 1 _2oxoadp_c + 1 akg_m = 1 _2oxoadp_m + 1 akg_c | 1 _2_Oxoadipate + 1 _2_Oxoglutarate = 1 _2_Oxoadipate + 1 _2_Oxoglutarate |
| **268** | _2AMADPTm | 1 L2aadp_c + 1 akg_m = 1 L2aadp_m + 1 akg_c | 1 L_2_Aminoadipate + 1 _2_Oxoglutarate = 1 L_2_Aminoadipate + 1 _2_Oxoglutarate |
| **269** | SMS | 1 crm_hs_c + 1 pchol_hs_c = 1 dag_hs_c + 1 sphmyln_hs_c | 1 ceramide_homosapiens + 1 Phosphatidylcholine_homosapiens = 1 diacylglycerol_homosapiens + 1 sphingomyelin_homosapiens |
| **270** | C3STDH1Pr | 1 _4mzym_int1_r + 1 nadp_r = 1 _4mzym_int2_r + 1 co2_r + 1 h_r + 1 nadph_r | 1 _4_Methylzymosterolintermediate1 + 1 Nicotinamideadeninedinucleotidephosphate = 1 _4_Methylzymosterolintermediate2 + 1 CO2 + 1 H+ + 1 Nicotinamideadeninedinucleotidephosphate_reduced |
| **271** | C4STMO2Pr | 1 _4mzym_int2_r + 1 nadp_r + 1 o2_r = 1 co2_r + 1 h_r + 1 nadph_r + 1 zym_int2_r | 1 _4_Methylzymosterolintermediate2 + 1 Nicotinamideadeninedinucleotidephosphate + 1 O2 = 1 CO2 + 1 H+ + 1 Nicotinamideadeninedinucleotidephosphate_reduced + 1 zymosterolintermediate2 |
| **272** | _3SPYRSP | 1 _3snpyr_c + 1 h2o_c = 1 h_c + 1 pyr_c + 1 so3_c | 1 _3_Sulfinopyruvate + 1 H2O = 1 H+ + 1 Pyruvate + 1 Sulfite |
| **273** | AASAD3m | 1 L2aadp6sa_m + 1 h2o_m + 1 nad_m = 1 L2aadp_m + 2 h_m + 1 nadh_m | 1 L_2_Aminoadipate6_semialdehyde + 1 H2O + 1 Nicotinamideadeninedinucleotide = 1 L_2_Aminoadipate + 2 H+ + 1 Nicotinamideadeninedinucleotide_reduced |
| **274** | ETF | 1 etfox_m + 1 fadh2_m = 1 etfrd_m + 1 fad_m | 1 Electrontransferflavoproteinoxidized + 1 Flavinadeninedinucleotidereduced = 1 Electrontransferflavoproteinreduced + 1 Flavinadeninedinucleotideoxidized |
| **275** | ETFQO | 1 etfrd_m + 1 q10_m = 1 etfox_m + 1 q10h2_m | 1 Electrontransferflavoproteinreduced + 1 Ubiquinone_10 = 1 Electrontransferflavoproteinoxidized + 1 Ubiquinol_10 |
| **276** | ASNt4 | 1 asn_L_e + 1 na1_e = 1 asn_L_c + 1 na1_c | 1 L_Asparagine + 1 Sodium = 1 L_Asparagine + 1 Sodium |
| **277** | ARGtiDF | 1 arg_L_e = 1 arg_L_c | 1 L_Arginine = 1 L_Arginine |
| **278** | HIStiDF | 1 his_L_e = 1 his_L_c | 1 L_Histidine = 1 L_Histidine |
| **279** | LYStiDF | 1 lys_L_e = 1 lys_L_c | 1 L_Lysine = 1 L_Lysine |
| **280** | FTHFDH | 1 _10fthf_c + 1 h2o_c + 1 nadp_c = 1 co2_c + 1 h_c + 1 nadph_c + 1 thf_c | 1 _10_Formyltetrahydrofolate + 1 H2O + 1 Nicotinamideadeninedinucleotidephosphate = 1 CO2 + 1 H+ + 1 Nicotinamideadeninedinucleotidephosphate_reduced + 1 _5_6_7_8_Tetrahydrofolate |
| **281** | FTCD | 1 _5forthf_c + 2 h_c = 1 methf_c + 1 nh4_c | 1 _5_Formiminotetrahydrofolate + 2 H+ = 1 _5_10_Methenyltetrahydrofolate + 1 Ammonium |
| **282** | GLCt1r | 1 glc_D_e = 1 glc_D_c | 1 D_Glucose = 1 D_Glucose |
| **283** | ASPt6 | 1 asp_L_e + 1 h_e + 1 k_c + 3 na1_e = 1 asp_L_c + 1 h_c + 1 k_e + 3 na1_c | 1 L_Aspartate + 1 H+ + 1 potassium + 3 Sodium = 1 L_Aspartate + 1 H+ + 1 potassium + 3 Sodium |
| **284** | PSDm_hs | 1 h_m + 1 ps_hs_m = 1 co2_m + 1 pe_hs_m | 1 H+ + 1 phosphatidylserine_homosapiens = 1 CO2 + 1 phosphatidylethanolamine_homosapiens |
| **285** | GLNCYSNaEx | 1 cys_L_c + 1 gln_L_e + 1 na1_e = 1 cys_L_e + 1 gln_L_c + 1 na1_c | 1 L_Cysteine + 1 L_Glutamine + 1 Sodium = 1 L_Cysteine + 1 L_Glutamine + 1 Sodium |
| **286** | GLUVESSEC | 1 atp_c + 1 glu_L_c + 1 h2o_c = 1 adp_c + 1 glu_L_e + 1 h_c + 1 pi_c | 1 ATP + 1 L_Glutamate + 1 H2O = 1 ADP + 1 L_Glutamate + 1 H+ + 1 Phosphate |
| **287** | LEUtec | 1 leu_L_e = 1 leu_L_c | 1 L_Leucine = 1 L_Leucine |
| **288** | ILEtec | 1 ile_L_e = 1 ile_L_c | 1 L_Isoleucine = 1 L_Isoleucine |
| **289** | VALtec | 1 val_L_e = 1 val_L_c | 1 L_Valine = 1 L_Valine |
| **290** | METtec | 1 met_L_e = 1 met_L_c | 1 L_Methionine = 1 L_Methionine |
| **291** | PHEtec | 1 phe_L_e = 1 phe_L_c | 1 L_Phenylalanine = 1 L_Phenylalanine |
| **292** | CYStec | 1 cys_L_e = 1 cys_L_c | 1 L_Cysteine = 1 L_Cysteine |
| **293** | NAt | 1 na1_e = 1 na1_c | 1 Sodium = 1 Sodium |
| **294** | UREAt5 | 1 h2o_e + 1 urea_e = 1 h2o_c + 1 urea_c | 1 H2O + 1 Urea = 1 H2O + 1 Urea |
| **295** | GLCt2r | 1 glc_D_e + 1 h_e = 1 glc_D_c + 1 h_c | 1 D_Glucose + 1 H+ = 1 D_Glucose + 1 H+ |
| **296** | DHORD9 | 1 dhor_S_c + 1 q10_m = 1 orot_c + 1 q10h2_m | 1 _S_Dihydroorotate + 1 Ubiquinone_10 = 1 Orotate + 1 Ubiquinol_10 |
| **297** | AMETt2m | 1 ahcys_m + 1 amet_c = 1 ahcys_c + 1 amet_m | 1 S_Adenosyl_L_homocysteine + 1 S_Adenosyl_L_methionine = 1 S_Adenosyl_L_homocysteine + 1 S_Adenosyl_L_methionine |
| **298** | SO4t4_2 | 2 na1_e + 1 so4_e = 2 na1_c + 1 so4_c | 2 Sodium + 1 Sulfate = 2 Sodium + 1 Sulfate |
| **299** | TYRt | 1 tyr_L_e = 1 tyr_L_c | 1 L_Tyrosine = 1 L_Tyrosine |
| **300** | TRPt | 1 trp_L_e = 1 trp_L_c | 1 L_Tryptophan = 1 L_Tryptophan |
| **301** | VALt5m | 1 val_L_c = 1 val_L_m | 1 L_Valine = 1 L_Valine |
| **302** | LEUt5m | 1 leu_L_c = 1 leu_L_m | 1 L_Leucine = 1 L_Leucine |
| **303** | ILEt5m | 1 ile_L_c = 1 ile_L_m | 1 L_Isoleucine = 1 L_Isoleucine |
| **304** | NH4t3r | 1 h_e + 1 nh4_c = 1 h_c + 1 nh4_e | 1 H+ + 1 Ammonium = 1 H+ + 1 Ammonium |
| **305** | FRDPtr | 1 frdp_x = 1 frdp_r | 1 Farnesyldiphosphate = 1 Farnesyldiphosphate |
| **306** | MMMm | 1 mmcoa_R_m = 1 succoa_m | 1 _R_Methylmalonyl_CoA = 1 Succinyl_CoA |
| **307** | G3PD2m | 1 fad_m + 1 glyc3p_c = 1 dhap_c + 1 fadh2_m | 1 Flavinadeninedinucleotideoxidized + 1 Glycerol3_phosphate = 1 Dihydroxyacetonephosphate + 1 Flavinadeninedinucleotidereduced |
| **308** | CHSTEROLt2 | 1 chsterol_r = 1 chsterol_m | 1 Cholesterol = 1 Cholesterol |
| **309** | CHSTEROLt3 | 1 chsterol_c = 1 chsterol_m | 1 Cholesterol = 1 Cholesterol |
| **310** | ATPtx | 1 atp_c = 1 atp_x | 1 ATP = 1 ATP |
| **311** | ADPtx | 1 adp_c = 1 adp_x | 1 ADP = 1 ADP |
| **312** | ARTCOAL1 | 1 Rtotal_c + 1 coa_c = 1 Rtotalcoa_c | 1 Rtotal + 1 CoenzymeA = 1 RtotalCoenzymeA |
| **313** | ARTPLM1 | 1 Rtotalcoa_c = 1 pmtcoa_c | 1 RtotalCoenzymeA = 1 Palmitoyl_CoA_n_C16_0CoA |
| **314** | ARTPLM2 | 1 Rtotal2coa_c = 1 pmtcoa_c | 1 Rtotal2coenzymeA = 1 Palmitoyl_CoA_n_C16_0CoA |
| **315** | NaKt | 1 atp_c + 1 h2o_c + 2 k_e + 3 na1_c = 1 adp_c + 1 h_c + 2 k_c + 3 na1_e + 1 pi_c | 1 ATP + 1 H2O + 2 potassium + 3 Sodium = 1 ADP + 1 H+ + 2 potassium + 3 Sodium + 1 Phosphate |
| **316** | Htr | 1 h_c = 1 h_r | 1 H+ = 1 H+ |
| **317** | FADtru | 1 fad_r = 1 fad_c | 1 Flavinadeninedinucleotideoxidized = 1 Flavinadeninedinucleotideoxidized |
| **318** | NADPHtru | 1 nadph_c = 1 nadph_r | 1 Nicotinamideadeninedinucleotidephosphate_reduced = 1 Nicotinamideadeninedinucleotidephosphate_reduced |
| **319** | NADPtru | 1 nadp_r = 1 nadp_c | 1 Nicotinamideadeninedinucleotidephosphate = 1 Nicotinamideadeninedinucleotidephosphate |
| **320** | FADH2tru | 1 fadh2_c = 1 fadh2_r | 1 Flavinadeninedinucleotidereduced = 1 Flavinadeninedinucleotidereduced |
| **321** | FORtr | 1 for_c = 1 for_r | 1 Formate = 1 Formate |
| **322** | PPItx | 1 ppi_c = 1 ppi_x | 1 Diphosphate = 1 Diphosphate |
| **323** | PItx | 1 pi_c = 1 pi_x | 1 Phosphate = 1 Phosphate |
| **324** | Htx | 1 h_c = 1 h_x | 1 H+ = 1 H+ |
| **325** | NADPHtxu | 1 nadph_c = 1 nadph_x | 1 Nicotinamideadeninedinucleotidephosphate_reduced = 1 Nicotinamideadeninedinucleotidephosphate_reduced |
| **326** | NADPtxu | 1 nadp_x = 1 nadp_c | 1 Nicotinamideadeninedinucleotidephosphate = 1 Nicotinamideadeninedinucleotidephosphate |
| **327** | HMGCOAtx | 1 hmgcoa_c = 1 hmgcoa_x | 1 Hydroxymethylglutaryl_CoA = 1 Hydroxymethylglutaryl_CoA |
| **328** | MMTSADm | 1 _2mop_m + 1 coa_m + 1 nad_m = 1 h_m + 1 mmcoa_R_m + 1 nadh_m | 1 _2_Methyl_3_oxopropanoate + 1 CoenzymeA + 1 Nicotinamideadeninedinucleotide = 1 H+ + 1 _R_Methylmalonyl_CoA + 1 Nicotinamideadeninedinucleotide_reduced |
| **329** | DSAT | 1 Rtotalcoa_c + 1 sphgn_c = 1 coa_c + 1 dhcrm_hs_c + 1 h_c | 1 RtotalCoenzymeA + 1 Sphinganine = 1 CoenzymeA + 1 dihydroceramide_homosapiens + 1 H+ |
| **330** | _3SALATAi | 1 _3sala_c + 1 akg_c + 1 h_c = 1 _3snpyr_c + 1 glu_L_c | 1 _3_Sulfino_L_alanine + 1 _2_Oxoglutarate + 1 H+ = 1 _3_Sulfinopyruvate + 1 L_Glutamate |
| **331** | DHPR2 | 1 _5mthf_c + 1 dhbpt_c = 1 h_c + 1 mlthf_c + 1 thbpt_c | 1 _5_Methyltetrahydrofolate + 1 _6_7_Dihydrobiopterin = 1 H+ + 1 _5_10_Methylenetetrahydrofolate + 1 Tetrahydrobiopterin |
| **332** | THBPT4ACAMDASE | 1 thbpt4acam_c = 1 dhbpt_c + 1 h2o_c | 1 Tetrahydrobiopterin_4a_carbinolamine = 1 _6_7_Dihydrobiopterin + 1 H2O |
| **333** | PHETHPTOX2 | 1 o2_c + 1 phe_L_c + 1 thbpt_c = 1 thbpt4acam_c + 1 tyr_L_c | 1 O2 + 1 L_Phenylalanine + 1 Tetrahydrobiopterin = 1 Tetrahydrobiopterin_4a_carbinolamine + 1 L_Tyrosine |
| **334** | GPAM_hs | 1 Rtotalcoa_c + 1 glyc3p_c = 1 alpa_hs_c + 1 coa_c | 1 RtotalCoenzymeA + 1 Glycerol3_phosphate = 1 lysophosphatidicacid_homosapiens + 1 CoenzymeA |
| **335** | AGPAT1 | 1 Rtotal2coa_c + 1 alpa_hs_c = 1 coa_c + 1 pa_hs_c | 1 Rtotal2coenzymeA + 1 lysophosphatidicacid_homosapiens = 1 CoenzymeA + 1 phosphatidicacid_homosapiens |
| **336** | PSFLIPm | 1 atp_c + 1 h2o_c + 1 ps_hs_c = 1 adp_c + 1 h_c + 1 pi_c + 1 ps_hs_m | 1 ATP + 1 H2O + 1 phosphatidylserine_homosapiens = 1 ADP + 1 H+ + 1 Phosphate + 1 phosphatidylserine_homosapiens |
| **337** | PCFLOPm | 1 atp_c + 1 h2o_c + 1 pchol_hs_m = 1 adp_c + 1 h_c + 1 pchol_hs_c + 1 pi_c | 1 ATP + 1 H2O + 1 Phosphatidylcholine_homosapiens = 1 ADP + 1 H+ + 1 Phosphatidylcholine_homosapiens + 1 Phosphate |
| **338** | SULFOX | 2 ficytC_m + 1 h2o_c + 1 so3_c = 2 focytC_m + 2 h_c + 1 so4_c | 2 Ferricytochromec + 1 H2O + 1 Sulfite = 2 FerrocytochromeC + 2 H+ + 1 Sulfate |
| **339** | PETOHMm_hs | 3 amet_m + 1 pe_hs_m = 3 ahcys_m + 3 h_m + 1 pchol_hs_m | 3 S_Adenosyl_L_methionine + 1 phosphatidylethanolamine_homosapiens = 3 S_Adenosyl_L_homocysteine + 3 H+ + 1 Phosphatidylcholine_homosapiens |
| **340** | SPHMDAc | 1 h2o_c + 1 sphmyln_hs_c = 1 Rtotal_c + 1 spc_hs_c | 1 H2O + 1 sphingomyelin_homosapiens = 1 Rtotal + 1 sphingosylphosphorylcholine_homosapiens |
| **341** | _2MCITt | 1 _2mcit_c = 1 _2mcit_e | 1 _2_Methylcitrate = 1 _2_Methylcitrate |
| **342** | EX_2mcit_e | 1 _2mcit_e = 1 _2mcit_b | 1 _2_Methylcitrate = 1 _2_Methylcitrate |
| **343** | O2Stm | 1 o2s_c = 1 o2s_m | 1 Superoxideanion = 1 Superoxideanion |
| **344** | H2O2tm | 1 h2o2_c = 1 h2o2_m | 1 Hydrogenperoxide = 1 Hydrogenperoxide |
| **345** | PE_HStm | 1 pe_hs_c = 1 pe_hs_m | 1 phosphatidylethanolamine_homosapiens = 1 phosphatidylethanolamine_homosapiens |
| **346** | BIO_ala | 1 ala_L_c + 4.3 atp_c + 4.3 h2o_c = 1 ala_L_bio + 4.3 adp_c + 4.3 h_c + 4.3 pi_c | 1 L_Alanine + 4.3 ATP + 4.3 H2O = 1 L_Alanine + 4.3 ADP + 4.3 H+ + 4.3 Phosphate |
| **347** | BIO_arg | 1 arg_L_c + 4.3 atp_c + 4.3 h2o_c = 1 arg_L_bio + 4.3 adp_c + 4.3 h_c + 4.3 pi_c | 1 L_Arginine + 4.3 ATP + 4.3 H2O = 1 L_Arginine + 4.3 ADP + 4.3 H+ + 4.3 Phosphate |
| **348** | BIO_asn | 1 asn_L_c + 4.3 atp_c + 4.3 h2o_c = 1 asn_L_bio + 4.3 adp_c + 4.3 h_c + 4.3 pi_c | 1 L_Asparagine + 4.3 ATP + 4.3 H2O = 1 L_Asparagine + 4.3 ADP + 4.3 H+ + 4.3 Phosphate |
| **349** | BIO_asp | 1 asp_L_c + 4.3 atp_c + 4.3 h2o_c = 1 asp_L_bio + 4.3 adp_c + 4.3 h_c + 4.3 pi_c | 1 L_Aspartate + 4.3 ATP + 4.3 H2O = 1 L_Aspartate + 4.3 ADP + 4.3 H+ + 4.3 Phosphate |
| **350** | BIO_cys | 1 cys_L_c + 4.3 atp_c + 4.3 h2o_c = 1 cys_L_bio + 4.3 adp_c + 4.3 h_c + 4.3 pi_c | 1 L_Cysteine + 4.3 ATP + 4.3 H2O = 1 L_Cysteine + 4.3 ADP + 4.3 H+ + 4.3 Phosphate |
| **351** | BIO_gln | 1 gln_L_c + 4.3 atp_c + 4.3 h2o_c = 1 gln_L_bio + 4.3 adp_c + 4.3 h_c + 4.3 pi_c | 1 L_Glutamine + 4.3 ATP + 4.3 H2O = 1 L_Glutamine + 4.3 ADP + 4.3 H+ + 4.3 Phosphate |
| **352** | BIO_glu | 1 glu_L_c + 4.3 atp_c + 4.3 h2o_c = 1 glu_L_bio + 4.3 adp_c + 4.3 h_c + 4.3 pi_c | 1 L_Glutamate + 4.3 ATP + 4.3 H2O = 1 L_Glutamate + 4.3 ADP + 4.3 H+ + 4.3 Phosphate |
| **353** | BIO_gly | 1 gly_c + 4.3 atp_c + 4.3 h2o_c = 1 gly_bio + 4.3 adp_c + 4.3 h_c + 4.3 pi_c | 1 Glycine + 4.3 ATP + 4.3 H2O = 1 Glycine + 4.3 ADP + 4.3 H+ + 4.3 Phosphate |
| **354** | BIO_his | 1 his_L_c + 4.3 atp_c + 4.3 h2o_c = 1 his_L_bio + 4.3 adp_c + 4.3 h_c + 4.3 pi_c | 1 L_Histidine + 4.3 ATP + 4.3 H2O = 1 L_Histidine + 4.3 ADP + 4.3 H+ + 4.3 Phosphate |
| **355** | BIO_ile | 1 ile_L_c + 4.3 atp_c + 4.3 h2o_c = 1 ile_L_bio + 4.3 adp_c + 4.3 h_c + 4.3 pi_c | 1 L_Isoleucine + 4.3 ATP + 4.3 H2O = 1 L_Isoleucine + 4.3 ADP + 4.3 H+ + 4.3 Phosphate |
| **356** | BIO_leu | 1 leu_L_c + 4.3 atp_c + 4.3 h2o_c = 1 leu_L_bio + 4.3 adp_c + 4.3 h_c + 4.3 pi_c | 1 L_Leucine + 4.3 ATP + 4.3 H2O = 1 L_Leucine + 4.3 ADP + 4.3 H+ + 4.3 Phosphate |
| **357** | BIO_lys | 1 lys_L_c + 4.3 atp_c + 4.3 h2o_c = 1 lys_L_bio + 4.3 adp_c + 4.3 h_c + 4.3 pi_c | 1 L_Lysine + 4.3 ATP + 4.3 H2O = 1 L_Lysine + 4.3 ADP + 4.3 H+ + 4.3 Phosphate |
| **358** | BIO_met | 1 met_L_c + 4.3 atp_c + 4.3 h2o_c = 1 met_L_bio + 4.3 adp_c + 4.3 h_c + 4.3 pi_c | 1 L_Methionine + 4.3 ATP + 4.3 H2O = 1 L_Methionine + 4.3 ADP + 4.3 H+ + 4.3 Phosphate |
| **359** | BIO_phe | 1 phe_L_c + 4.3 atp_c + 4.3 h2o_c = 1 phe_L_bio + 4.3 adp_c + 4.3 h_c + 4.3 pi_c | 1 L_Phenylalanine + 4.3 ATP + 4.3 H2O = 1 L_Phenylalanine + 4.3 ADP + 4.3 H+ + 4.3 Phosphate |
| **360** | BIO_pro | 1 pro_L_c + 4.3 atp_c + 4.3 h2o_c = 1 pro_L_bio + 4.3 adp_c + 4.3 h_c + 4.3 pi_c | 1 L_Proline + 4.3 ATP + 4.3 H2O = 1 L_Proline + 4.3 ADP + 4.3 H+ + 4.3 Phosphate |
| **361** | BIO_ser | 1 ser_L_c + 4.3 atp_c + 4.3 h2o_c = 1 ser_L_bio + 4.3 adp_c + 4.3 h_c + 4.3 pi_c | 1 L_Serine + 4.3 ATP + 4.3 H2O = 1 L_Serine + 4.3 ADP + 4.3 H+ + 4.3 Phosphate |
| **362** | BIO_thr | 1 thr_L_c + 4.3 atp_c + 4.3 h2o_c = 1 thr_L_bio + 4.3 adp_c + 4.3 h_c + 4.3 pi_c | 1 L_Threonine + 4.3 ATP + 4.3 H2O = 1 L_Threonine + 4.3 ADP + 4.3 H+ + 4.3 Phosphate |
| **363** | BIO_trp | 1 trp_L_c + 4.3 atp_c + 4.3 h2o_c = 1 trp_L_bio + 4.3 adp_c + 4.3 h_c + 4.3 pi_c | 1 L_Tryptophan + 4.3 ATP + 4.3 H2O = 1 L_Tryptophan + 4.3 ADP + 4.3 H+ + 4.3 Phosphate |
| **364** | BIO_tyr | 1 tyr_L_c + 4.3 atp_c + 4.3 h2o_c = 1 tyr_L_bio + 4.3 adp_c + 4.3 h_c + 4.3 pi_c | 1 L_Tyrosine + 4.3 ATP + 4.3 H2O = 1 L_Tyrosine + 4.3 ADP + 4.3 H+ + 4.3 Phosphate |
| **365** | BIO_val | 1 val_L_c + 4.3 atp_c + 4.3 h2o_c = 1 val_L_bio + 4.3 adp_c + 4.3 h_c + 4.3 pi_c | 1 L_Valine + 4.3 ATP + 4.3 H2O = 1 L_Valine + 4.3 ADP + 4.3 H+ + 4.3 Phosphate |
| **366** | BIO_atp | 1 atp_c + 1 h2o_c = 1 atp_bio + 1 h_c + 1 ppi_c | 1 ATP + 1 H2O = 1 ATP + 1 H+ + 1 Diphosphate |
| **367** | BIO_gtp | 1 gtp_c + 1 h2o_c = 1 gtp_bio + 1 h_c + 1 ppi_c | 1 GTP + 1 H2O = 1 GTP + 1 H+ + 1 Diphosphate |
| **368** | BIO_ctp | 1 ctp_c + 1 h2o_c = 1 ctp_bio + 1 h_c + 1 ppi_c | 1 CTP + 1 H2O = 1 CTP + 1 H+ + 1 Diphosphate |
| **369** | BIO_utp | 1 utp_c + 1 h2o_c = 1 utp_bio + 1 h_c + 1 ppi_c | 1 UTP + 1 H2O = 1 UTP + 1 H+ + 1 Diphosphate |
| **370** | BIO_datp | 1 datp_c + 1 h2o_c = 1 datp_bio + 1 h_c + 1 ppi_c | 1 dATP + 1 H2O = 1 dATP + 1 H+ + 1 Diphosphate |
| **371** | BIO_dgtp | 1 dgtp_c + 1 h2o_c = 1 dgtp_bio + 1 h_c + 1 ppi_c | 1 dGTP + 1 H2O = 1 dGTP + 1 H+ + 1 Diphosphate |
| **372** | BIO_dctp | 1 dctp_c + 1 h2o_c = 1 dctp_bio + 1 h_c + 1 ppi_c | 1 dCTP + 1 H2O = 1 dCTP + 1 H+ + 1 Diphosphate |
| **373** | BIO_dttp | 1 dttp_c + 1 h2o_c = 1 dttp_bio + 1 h_c + 1 ppi_c | 1 dTTP + 1 H2O = 1 dTTP + 1 H+ + 1 Diphosphate |
| **374** | BIO_maint | 1 atp_c + 1 h2o_c = 1 adp_c + 1 h_c + 1 pi_c | 1 ATP + 1 H2O = 1 ADP + 1 H+ + 1 Phosphate |
| **375** | BIO_chol | 1 chsterol_c = 1 chsterol_bio | 1 Cholesterol = 1 Cholesterol |
| **376** | BIO_pe | 1 pe_hs_c = 1 pe_hs_bio | 1 phosphatidylethanolamine_homosapiens = 1 phosphatidylethanolamine_homosapiens |
| **377** | BIO_pc | 1 pchol_hs_c = 1 pchol_hs_bio | 1 Phosphatidylcholine_homosapiens = 1 Phosphatidylcholine_homosapiens |
| **378** | BIO_sphingo | 1 spc_hs_c = 1 spc_hs_bio | 1 sphingosylphosphorylcholine_homosapiens = 1 sphingosylphosphorylcholine_homosapiens |
| **379** | BIO_cardiolipin | 1 clpn_hs_c = 1 clpn_hs_bio | 1 cardiolipin_homosapiens = 1 cardiolipin_homosapiens |
| **380** | BIO_ps | 1 ps_hs_c = 1 ps_hs_bio | 1 phosphatidylserine_homosapiens = 1 phosphatidylserine_homosapiens |
| **381** | BIO_pg | 1 pglyc_hs_c = 1 pglyc_hs_bio | 1 phosphatidylglycerol_homosapiens = 1 phosphatidylglycerol_homosapiens |
| **382** | EX_GTX_e | 1 GTX_e = 1 GTX_b | 1 Glutamax = 1 Glutamax |
| **383** | GTX_hydrolysis | 1 GTX_e = 1 gln_L_e + 1 ala_L_e | 1 Glutamax = 1 L_Glutamine + 1 L_Alanine |
| **384** | BIO_mab | 16 ala_L_c + 13 arg_L_c + 16 asn_L_c + 15 asp_L_c + 8 cys_L_c + 13 gln_L_c + 21 glu_L_c + 24 gly_c + 8 his_L_c + 6 ile_L_c + 28 leu_L_c + 23 lys_L_c + 5 met_L_c + 11 phe_L_c + 27 pro_L_c + 36 ser_L_c + 24 thr_L_c + 6 trp_L_c + 15 tyr_L_c + 31 val_L_c + 1487.8 atp_c + 1487.8 h2o_c = 1487.8 adp_c + 1487.8 h_c + 1487.8 pi_c + 1 mab_bio | 16 L_Alanine + 13 L_Arginine + 16 L_Asparagine + 15 L_Aspartate + 8 L_Cysteine + 13 L_Glutamine + 21 L_Glutamate + 24 Glycine + 8 L_Histidine + 6 L_Isoleucine + 28 L_Leucine + 23 L_Lysine + 5 L_Methionine + 11 L_Phenylalanine + 27 L_Proline + 36 L_Serine + 24 L_Threonine + 6 L_Tryptophan + 15 L_Tyrosine + 31 L_Valine + 1487.8 ATP + 1487.8 H2O = 1487.8 ADP + 1487.8 H+ + 1487.8 Phosphate + 1 Antibody |
